# TRIM28 represses renal cell carcinoma cell proliferation by inhibiting TFE3/KDM6A-regulated autophagy

**DOI:** 10.1016/j.jbc.2023.104621

**Published:** 2023-03-18

**Authors:** Tanjing Song, Suli Lv, Xianyun Ma, Xuefeng Zhao, Li Fan, Qingli Zou, Neng Li, Yingying Yan, Wen Zhang, Lidong Sun

**Affiliations:** 1 Department of Biochemistry and Molecular Biology, School of Basic Medicine, Tongji Medical College, Huazhong University of Science and Technology, Wuhan, Hubei, China; 2 Cell Architecture Research Institute, Huazhong University of Science and Technology, Wuhan, Hubei, China; 3 Wuhan Children's Hospital, Tongji Medical College, Huazhong University of Science and Technology, Wuhan, Hubei, China

**Keywords:** TRIM28, TFE3, KDM6A, autophagy, kidney cancer, histone methylation, ubiquitination

## Abstract

Autophagy plays a pivotal role in physiology and pathophysiology, including cancer. Mechanisms of autophagy dysregulation in cancer remain elusive. Loss of function of TRIM28, a multifunction protein, is seen in familial kidney malignancy, but the mechanism by which TRIM28 contributes to the etiology of kidney malignancy is unclear. In this study, we show TRIM28 retards kidney cancer cell proliferation through inhibiting autophagy. Mechanistically, we find TRIM28 promotes ubiquitination and proteasome-mediated degradation of transcription factor TFE3, which is critical for autophagic gene expression. Genetic activation of TFE3 due to gene fusion is known to cause human kidney malignancy, but whether and how transcription activation by TFE3 involves chromatin changes is unclear. Here, we find another mode of TFE3 activation in human renal carcinoma. We find that TFE3 is constitutively localized to the cell nucleus in human and mouse kidney cancer, where it increases autophagic gene expression and promotes cell autophagy as well as proliferation. We further uncover that TFE3 interacts with and recruits histone H3K27 demethylase KDM6A for autophagic gene upregulation. We reveal that KDM6A contributes to expression of TFE3 target genes through increasing H3K4me3 rather than demethylating H3K27. Collectively, in this study, we identify a functional TRIM28–TFE3–KDM6A signal axis, which plays a critical role in kidney cancer cell autophagy and proliferation.

Kidney cancer is one of the major cancer types worldwide with over 400,000 new cases each year, with renal cell carcinoma (RCC) being the most common type, which includes clear cell carcinoma, papillary cell carcinoma, and chromophobe cell carcinoma (1, 2). Genetic aberration including VHL mutation has been studied in kidney cancer. But the mechanisms of many other kidney cancer-driver genes are understudied.

TRIM28, a multifunction protein, was reported to play a role in the etiology of different cancer types (3). TRIM28 exhibits divergent function in different cancer types. While most reports showed TRIM28 promotes tumor progression, some reported otherwise (3, 4, 5, 6, 7, 8). For example, TRIM28 was reported to promote cell proliferation in breast cancer and inhibit cell proliferation in early-stage lung cancer (5, 9). Besides the diverse biological contexts in different cancers, diversity of TRIM28's biochemical activities also contributed to the complexity. TRIM28 can associate with SETDB1 and KRAB-family transcription factors to regulate transcription. In addition, TRIM28 is involved in DNA damage repair and transcription elongation (3, 10). Besides, TRIM28 has intrinsic E3 ligase activity for both SUMO and Ubiquitin (11, 12). TRIM28 ubiquitinates various substrates either dependent on or independent of Melanoma antigen gene (MAGE) (8, 12, 13, 14, 15, 16). *Via* these pleiotropic activities, TRIM28 plays important roles in physiology and pathophysiology. Genetic evidence shows loss of TRIM28 gives rise to familiar Wilms tumor, a renal blastoma affecting children, which strongly indicates TRIM28 has a distinct role in kidney malignancy (17, 18, 19, 20). Yet, the mechanism underlying TRIM28 function in kidney malignancy is not clear.

Autophagy, a critical process regulating cellular homeostasis, plays bipartite roles in cancer. Autophagy inhibits oncogenesis through maintaining physiological homeostasis (21, 22). However, autophagy also empowers established cancer cells. Increased autophagy has been linked to cancer progression and therapeutic resistance in many cancers (23). As an important process for cellular homeostasis, autophagy is under intricate regulation. Regulation of gene expression plays a pivotal role in control of autophagy, as established during the past few years (24). Epigenetic machinery and transcription factors regulate the expression of autophagic/lysosomal genes (25). Among transcription factors that positively regulate autophagic gene transcription, MITF family members, TFE3, TFEB, and MITF, have gained extensive attention (25). These three transcription factors can bind directly to evolutionarily conserved elements in autophagic/lysosomal gene promoters to promote expression of these genes (25, 26). Importantly, these three transcription factors mediate cellular autophagy response to starvation. Physiologically, they localize to the cytoplasm under replete condition and translocate to nucleus under starvation (26, 27). Consistent with increased autophagy in cancers, dysregulation of MITF family members is well recognized in cancer, which associates with abnormality in the autophagy-lysosome pathway. For example, their abnormally constitutive location to the nucleus was reported previously in pancreatic adenocarcinoma (28), which contributes to cancer malignancy. Intriguingly, among all organs, kidney has a long-established distinct connection with TFE3 function. TFE3 activation due to gene fusion constitutes about 1% of RCC cases (29, 30). But the function and regulatory mechanisms of TFE3 in RCC is not clear.

Intrigued by the genetic evidence of TRIM28 loss of function in kidney malignancy, we set out to investigate the role of TRIMI28 in kidney cancer in this study. We find TRIM28 suppresses RCC cell growth, which can be attributed to retarded autophagy. Mechanistically, we uncover TRIM28 promotes ubiquitination and proteasome-mediated degradation of TFE3. We further find that TFE3 is constitutively localized to nuclei in RCC cells and tumor samples. Consistently, TFE3 plays a significant role in RCC cell autophagy and proliferation. We go on to explore the potential impact of TFE3 on chromatin status of target genes. We find TFE3 interacts with and recruits KDM6A to target genes. KDM6A is a histone H3K27 demethylase, but it can also function independent of this activity (31, 32, 33). Knocking down KDM6A significantly inhibits RCC cell autophagy and autophagic gene expression. We also reveal KDM6A exerts this function independent of its H3K27 demethylase activity but rather through increasing local MLL3/4-mediated H3K4me3.

## Results

### TRIM28 inhibits RCC cell proliferation

**Figure 1:**
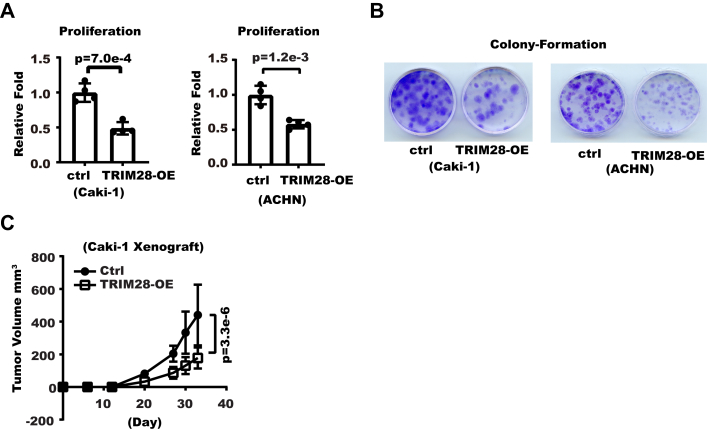
**TRIM28 inhibits renal cell carcinoma cell proliferation.***A*, TRIM28 was overexpressed in Caki-1 or ACHN cells. Cell proliferation was measured with cell counting. Shown are relative proliferation fold over 7 days. Error bars denote standard deviation of four biological replicates. *p* Values were calculated from Student's *t* test. *B*, a total of 1000 control or TRIM28-OE Caki-1 and ACHN cells were seeded into 3.5-cm dishes. Fourteen days later, cell colonies were fixed and stained with crystal violet as shown. *C*, a total of 5∗10∧6 control or TRIM28-OE Caki-1 cells were inoculated to the flanks of immunodeficient NOG mice. Shown are the tumor growth curve. *p* Value was calculated from two-way ANOVA.

We first examined the relation between TRIM28 and RCC patient survival with kidney cancer datasets from the Clinical Proteomic Tumor Analysis Consortium (CPTAC) and The Cancer Genome Atlas (TCGA) projects. Both datasets indicated higher TRIM28 protein (CPTAC) and mRNA (TCGA) levels correlate with better overall survival in patients with clear cell renal carcinoma (Fig. S1, *A* and *B*). Next, we set out to examine the effect of TRIM28 on RCC cell proliferation with both cell counting and clonogenesis assay. As Western blot showed all three RCC cell lines that we examined expressed lower TRIM28 than 293T cells (Fig. S1*C*), we then introduced exogenous TRIM28 into Caki-1 and ACHN cells, which expressed even lower TRIM28 than 786-O. The result showed overexpressing TRIM28 significantly inhibited cell proliferation (Fig. 1, *A* and *B*). To determine whether TRIM28 had a similar effect on RCC cell proliferation in a mouse model, we then inoculated control and TRIM28-Overexpression (OE) Caki-1 cells to immunodeficient mice. The result showed TRIM28 overexpression reduced tumor growth (Fig. 1*C*). Collectively, these data showed TRIM28 represses RCC proliferation.

### TRIM28 inhibits RCC cell proliferation through retarded autophagy

**Figure 2:**
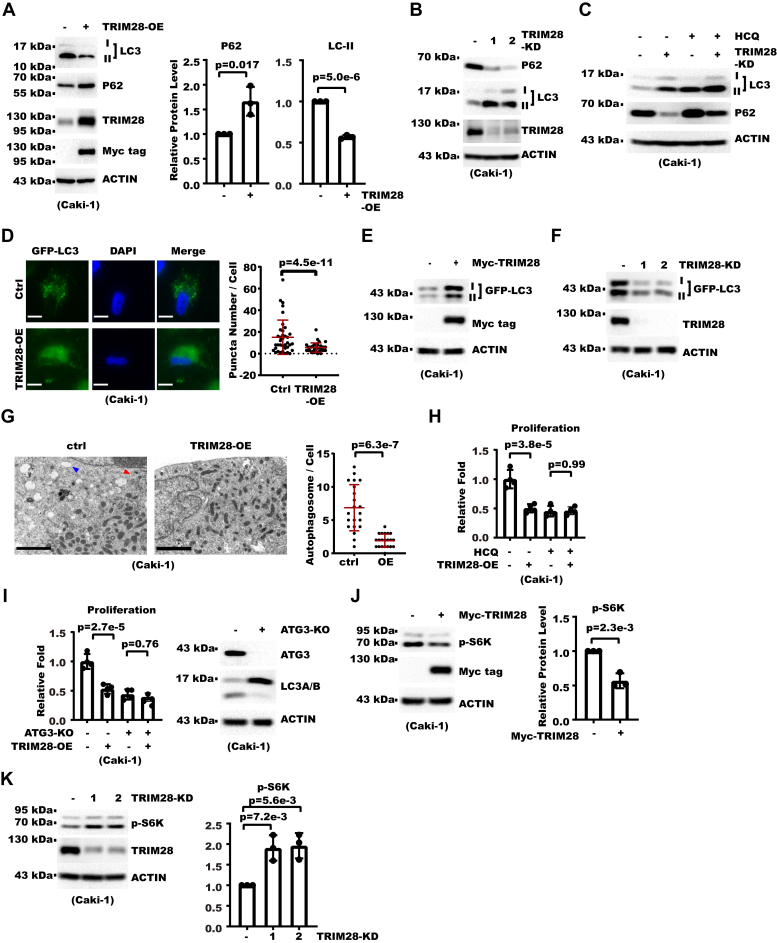
**TRIM28 inhibits renal cell carcinoma cell proliferation through retarded autophagy.***A*, Myc-TRIM28 was overexpressed by lentivirus transduction to get TRIM28 overexpression (OE) cells. Whole cell extract (WCE) of control and TRIM28-OE Caki-1 cells were analyzed with Western blot (WB). Shown on the *left* are WB results. Shown on the *right* is densitometry analysis for P62 and LC3-II levels from three biological replicates. Level shown represents the relative ratio between P62 or LC3-II and ACTIN from the same sample. Error bars denote standard deviation, and *p* values were calculated from Student's *t* test. *B*, shown are WB results for WCE of control and TRIM28-Knockdown (KD) Caki-1 cells. *C*, control or TRIM28-KD Caki-1 cells were treated with 20 μM HCQ for 8 h. WCE were then analyzed with WB as indicated. *D*, Myc-tagged TRIM28 was overexpressed in Caki-1 cells stably expressing GFP-LC3. Cells were fixed and nuclei were counterstained with DAPI. Shown on the *left* are cell photos from fluorescent microscopy (the scale bar represents 10 μm). Shown on the *right* is statistical analysis for number of GFP-LC3 puncta in each cell with mean and standard deviation shown in *red horizontal bars* (37 cells in Ctrl, 33 cells in OE). *p* Value was calculated from Student's *t* test. *E*, Myc-tagged TRIM28 were overexpressed in Caki-1 cells stably expressing GFP-LC3. WCE was then analyzed with WB as indicated. *F*, TRIM28 was knocked down in Caki-1 cells stably expressing GFP-LC3. WCE were then analyzed with WB as indicated. *G*, control and TRIM28-OE Caki-1 cells were analyzed with transmission electron microscopy. Shown on the *left* are representative pictures where the *red arrowhead* denotes autophagosome and the *blue arrowhead* denotes lysosome (the scale bar represents 2 μm). Shown on the *right* is statistical analysis for number of autophagosomes and autolysosomes in each photo with mean and standard deviation shown in *red horizontal bars* (21 cells in each group). *p* Value was calculated from Student's *t* test. *H*, control or TRIM28-OE Caki-1 cells were treated with 20 μM HCQ for 7 days. Shown are relative proliferation fold over the same period as examined by cell counting. *I*, Myc-tagged TRIM28 was overexpressed in control or ATG3-KO cells. Cell proliferation was analyzed with cell counting, and WCE was analyzed with WB. Shown on the *left* is relative proliferation fold over 7 days. Shown on the *right* are WB results. *J*, WCE of control and TRIM28-OE Caki-1 cells were analyzed with WB. Shown on the *left* are blot images. Shown on the *right* is densitometry analysis for p-S6K level from three biological replicates. p-S6K level is presented as the relative ratio between p-S6K and ACTIN. Error bars denote standard deviation of three technical replicates. *p* Values were calculated from Student's *t* test. *K*, WCE of control and TRIM28-KD Caki-1 cells were analyzed with WB. Shown on the *left* are blot images. Shown on the *right* is densitometry analysis for p-S6K level from three biological replicates. p-S6K level is presented as the relative ratio between p-S6K and ACTIN. Error bars denote standard deviation of three technical replicates. *p* Values were calculated from Student's *t* test.

When culturing TRIM28-OE RCC cells, we serendipitously noticed that they were more sensitive to starvation than control cells, which indicated that TRIM28 might regulate autophagy in RCC cells. LC3, the mammalian homologue of yeast ATG8, undergoes lipidation during autophagy and integrates into the autophagosome membrane (34). Another protein, P62, serves as a cargo adaptor during autophagy and itself gets degraded with the cargo in the lysosome. As a result, LC3 lipidation and P62 protein level are widely used as markers for autophagic activity (35). So we checked whether TRIM28 affected LC3 lipidation and P62 protein level. We found overexpressing TRIM28 decreased LC3-II (the lipidated form) and increased P62, both markers indicating decreased autophagy (Figs. 2*A* and S2*A*). On the contrary, knocking down TRIM28 gave the opposite effect (Fig. 2*B*). We then treated cells with lysosomal inhibitor hydroxychloroquine (HCQ) and found it increased LC3 and P62 accumulation in TRIM28-Knockdown (KD) cells, indicating TRIM28-KD did not block the autophagic flow (Fig. 2*C*). As the autophagosome harbors concentrated lipidated LC3 on its membrane, LC3 immunostaining signal presents as puncta visible under microscope. As a result, LC3 puncta are widely used as a surrogate for the autophagosome. To further confirm that TRIM28 regulates autophagy, we examined its effect on the number of LC3 puncta. We first established Caki-1 and ACHN cell lines stably expressing GFP-LC3 under the control of an exogenous promoter. Afterward, we overexpressed TRIM28 in these cells and found the number of LC3B puncta was significantly decreased (Fig. 2*D*). In addition, TRIM28-OE also decreased degradation of exogenous GFP-LC3 while TRIM28-KD gave the opposite result, further supporting TRIM28 inhibited autophagic degradation (Figs. 2, *E* and *F* and S2*B*). While LC3 puncta serve as a marker for the autophagosome, transmission electron microscopy (TEM) renders the opportunity to directly observe the autophagosome. With TEM, we confirmed TRIM28-OE indeed decreased the number of autophagosome/autolysosome (Fig. 2*G*). In addition, TRIM28-OE cells displayed enlarged lysosome filled with undigested cargoes, which also indicated defect in lysosomal function (Fig. S2*C*) (28). To confirm, we then immunostained the lysosome marker LAMP1 and found the average size of lysosomes was indeed increased in TRIM28-OE cells (Fig. S2*D*). The above data showed that TRIM28 repressed RCC cell proliferation as well as the autophagy-lysosome pathway. We next investigated whether these two effects of TRIM28 were connected. We blocked the autophagy pathway through HCQ treatment or knocking out ATG3, a key protein for LC3 lipidation and autophagy (34). The results showed TRIM28-OE rendered little further effect on cell proliferation (Fig. 2, *H* and *I*). Mechanistically, autophagy was shown to maintain activity of mTOR pathway, which is a major signaling hub promoting cell growth (28, 36). To examine whether TRIM28 affected mTOR activity in RCC cells, we overexpressed or knocked down TRIM28 and then measured the level of phosphorylated S6K, which is a substrate of mTOR and widely used as indicator of mTOR activity. We found TRIM28 overexpression decreased p-S6K level while TRIM28 knockdown increased p-S6K level (Figs. 2, *J* and *K* and S2*E*). In summary, these data showed TRIM28 suppressed RCC cell proliferation through inhibiting autophagy.

### TRIM28 decreases autophagic gene expression through downregulating TFE3 protein level

**Figure 3:**
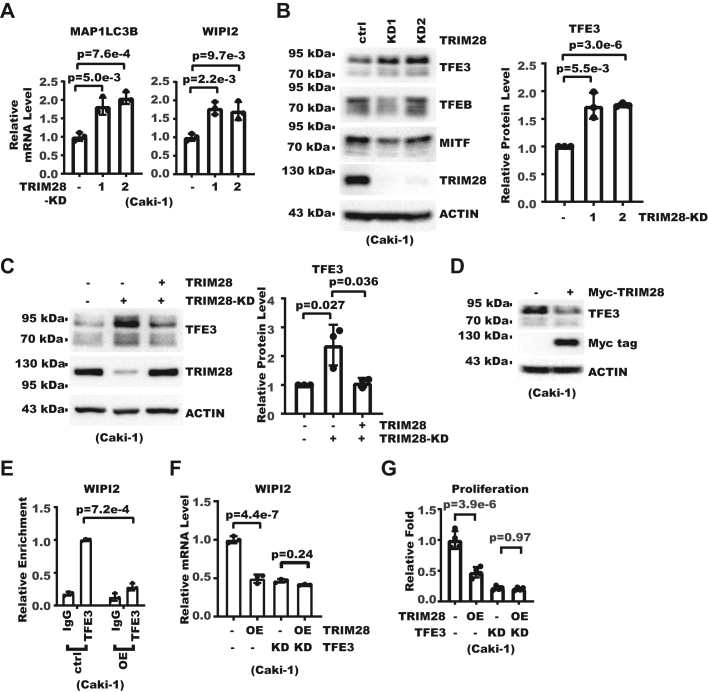
**TRIM28 decreases autophagic gene expression through downregulating TFE3 protein level.***A*, shown are real-time RT-PCR results for relative mRNA level of indicated genes in control and TRIM28-KD Caki-1 cells. Error bars denote standard deviation of three technical replicates. *p* Values were calculated from Student's *t* test. *B*, whole cell extracts (WCEs) of control and TRIM28-KD Caki-1 cells were analyzed with Western blot (WB). Shown on the *left* are blot images. Shown on the *right* is densitometry analysis for TFE3 level from WB of three biological replicates. TFE3 level is presented as the relative ratio between TFE3 and ACTIN. Error bars denote standard deviation of three technical replicates. *p* Values were calculated from Student's *t* test. *C*, WCE of control, TRIM28-KD, and TRIM28 rescue-expressing Caki-1 cells were analyzed with WB. Shown on the *left* are blot images. Shown on the *right* is densitometry analysis for TFE3 level from three biological replicates. TFE3 level is presented as the relative ratio between TFE3 and ACTIN. Error bars denote standard deviation of three technical replicates. *p* Values were calculated from Student's *t* test. *D*, WCE of control and TRIM28-OE Caki-1 cells were analyzed with WB. *E*, Myc-tagged TRIM28 was overexpressed in Caki-1 or ACHN cells. TFE3 enrichment on WIPI2 gene promoter was then analyzed with chromatin immunoprecipitation followed with real-time PCR. Error bars denote standard deviation of three technical replicates. *p* Values were calculated from Student's *t* test. *F*, TFE3 was knocked down in control or TRIM28-OE Caki-1 cells. Shown are real-time RT-PCR results for relative mRNA level of WIPI2. Error bars denote standard deviation of three technical replicates. *p* Values were calculated from one-way ANOVA. *G*, TFE3 was knocked down in control and TRIM28-OE Caki-1 cells. Cell proliferation was then analyzed with cell counting. Shown is relative proliferation fold over 7 days. Error bars denote standard deviation of four biological replicates. *p* Values were calculated from one-way ANOVA with correction for multiple comparison.

Next, we explored the mechanism how TRIM28 regulated autophagy in RCC. AMPKα, a known regulator of autophagy, was previously reported to be ubiquitinated by TRIM28 in certain cell types in a MAGE-dependent manner (13). However, in Caki-1, the AMPKα level was altered only modestly by TRIM28 (Fig. S3, *A* and *B*). On the other hand, we detected increased mRNA level of multiple autophagic genes upon TRIM28-KD (Figs. 3*A* and S3*C*). Interestingly, TFE3 was identified as a potential interaction partner of TRIM28 in a large-scale proteomic study (37). We confirmed the interaction between TRIM28 and TFE3 with coimmunoprecipitation (Fig. S3, *D* and *E*). To explore whether TRIM28 might affect TFE3, we first examined whether TRIM28 regulated TFE3 protein level. Western blot showed TRIM28-KD increased the protein level of TFE3 but not TFEB or MITF (Figs. 3*B* and S3*F*). The effect of TRIM28-KD on the TFE3 protein level could be reversed by introducing exogenous TRIM28, confirming the effect was specific rather than due to off-target effect (Fig. 3*C*). Consistently, TRIM28-OE decreased the TFE3 protein level (Fig. 3*D*). As Caki-1 and ACHN cells showed not only lower TRIM28 level but also higher ratio between TFE3 and TFEB/MITF (Fig. S3*G*), we performed mechanistic study mainly with these two cell lines hereafter. In light of the significant role of TFE3 in autophagy regulation, we next examined whether TFE3 mediated the effect of TRIM28 on autophagy in RCC cells. We performed chromatin immunoprecipitation (ChIP) analysis and found TRIM28 overexpression decreased enrichment of TFE3 at autophagic gene promoters, indicating TFE3 in RCC cells was active in binding target genes (Figs. 3*E* and S3*H*). Importantly, after TFE3 was knocked down, TRIM28 overexpression exhibited little further effect on autophagic gene expression (Figs. 3*F* and S3*I*). Consistently, our gene set enrichment analysis (GSEA) confirmed TRIM28 level negatively correlated with autophagy gene expression in those TCGA clear cell RCC samples where the TFE3 level was relatively higher than MITF and TFEB (Fig. S3*J*). These results suggested TRIM28 regulated autophagic gene expression through TFE3. Since we showed earlier autophagy underlay repressive effect of TRIM28 on RCC cell proliferation, we checked whether TFE3 also mediated effect of TRIM28 on RCC cell proliferation. We found, in TFE3-KD cells, TRIM28 overexpression rendered little further effect on cell proliferation (Fig. 3*G*). Collectively, these data showed TRIM28 regulated RCC cell autophagic gene expression and cell proliferation through TFE3.

### TRIM28 promotes ubiquitination-proteasome-mediated degradation of TFE3

**Figure 4:**
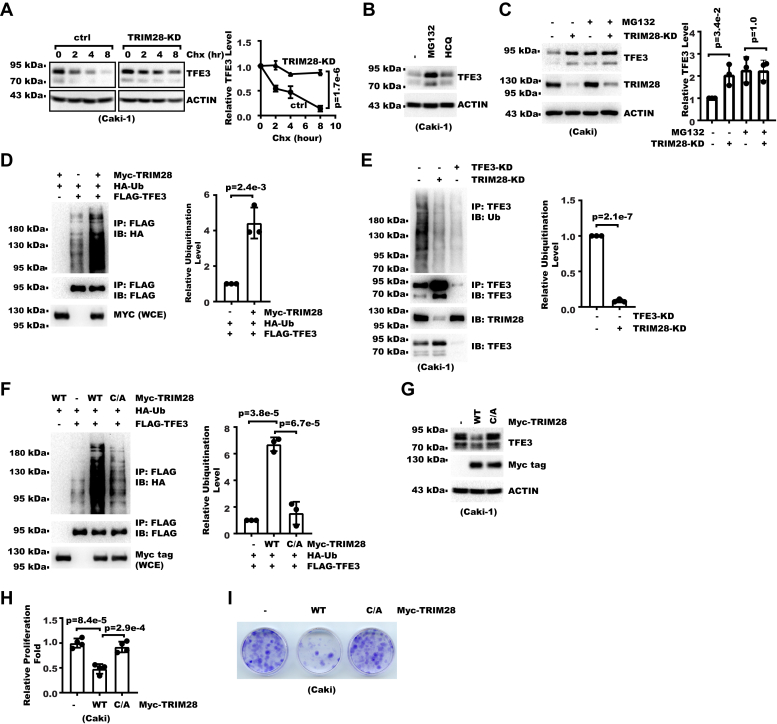
**TRIM28 promotes ubiquitination-proteasome-mediated degradation of TFE3.***A*, control and TRIM28-KD cells were treated with 25 μg/ml cycloheximide (Chx) for indicated time. Whole cell extracts (WCEs) were analyzed with Western blot (WB). Shown on the *left* are WB images. Shown on the *right* is densitometry analysis of TFE3 level from WB of three biological replicates. TFE3 level was presented as the relative ratio between TFE3 and ACTIN. Error bars denote standard deviation and *p* values were calculated from two-way ANOVA. *B*, Caki-1 and ACHN cells were treated with 25 μM MG132 or 20 μM HCQ for 8 h. WCEs were analyzed with WB. *C*, control or TRIM28-KD Caki-1 cells were treated with 25 μM MG132 for 8 h. WCEs were analyzed with WB. Shown on the *left* are blot images. Shown on the *right* is densitometry analysis of TFE3 levels from three biological replicates. TFE3 level is presented as the relative ratio between TFE3 and ACTIN. Error bars denote standard deviation of three biological replicates. *p* Values were calculated from one-way ANOVA. *D*, FLAG-TFE3, HA-Ub, and Myc-TRIM28 were cotransfected into 293T cells. Forty-eight hours later, cells were collected for immunoprecipitation WB analysis. Shown on the *left* are WB images. Shown on the *right* is densitometry analysis of TFE3 ubiquitination level from WB for three biological replicates. TFE3 ubiquitination level is presented as the relative ratio between ubiquitination and immunoprecipitated FLAG signal. Error bars denote standard deviation. *p* Values were calculated from Student's *t* test. *E*, endogenous TFE3 was immunoprecipitated from control or TRIM28-KD Caki-1 cells followed by WB analysis. Shown on the *left* are WB images. Shown on the *right* is densitometry analysis of TFE3 ubiquitination level from WB for three biological replicates. TFE3 ubiquitination level is presented as the relative ratio between ubiquitination and immunoprecipitated TFE3 signal. Error bars denote standard deviation of three biological replicates. *p* Values were calculated from Student's *t* test. *F*, FLAG-TFE3, HA-Ub, and Myc-TRIM28 (WT for wildtype or C/A for C65A/C68A) were cotransfected into 293T cells as indicated. Forty-eight hours later, cells were collected for immunoprecipitation WB analysis. Shown on the *left* are WB images. Shown on the *right* is densitometry analysis of FLAG-TFE3 ubiquitination level from WB for three biological replicates. FLAG-TFE3 ubiquitination level is presented as the relative ratio between ubiquitination and immunoprecipitated FLAG signal. Error bars denote standard deviation of three biological replicates. *p* Values were calculated from one-way ANOVA. *G*, Myc-tagged TRIM28 wildtype (WT) or inactive C65A/C68A mutant (C/A) was overexpressed in Caki-1. WCEs were analyzed with WB. *H*, proliferation of the same cells as in (*G*) was analyzed with cell counting. Shown are relative proliferation fold over 7 days. Error bars denote standard deviation of four biological replicates. *p* Values were calculated from one-way ANOVA. *I*, a total of 1000 TRIM28 wildtype (WT) or inactive C65A/C68A mutant (C/A) cells were seeded into 3.5-cm dishes. Fourteen days later, cell colonies were fixed and stained with crystal violet.

We next set out to determine how TRIM28 regulated TFE3. RT-PCR showed TRIM28-KD did not cause significant change in TFE3 mRNA (Fig. S4*A*). Instead, TRIM28-KD increased TFE3 protein stability after we treated cells with the protein synthesis inhibitor Cycloheximide (Figs. 4*A* and S4*B*). Lysosome and proteasome are two major routes for protein degradation. We found proteasome inhibitor MG132 but not lysosome inhibitor HCQ significantly increased TFE3 protein level, indicating the proteasome pathway played a major role in TFE3 protein stability control (Figs. 4*B* and S4*C*). After blocking proteasome with MG132, we found TRIM28-KD failed to further increase the TFE3 protein level (Figs. 4*C* and S4*D*), indicating it was the proteasome pathway that mediated regulation of TFE3 by TRIM28. As TFE3 activity was previously shown to be regulated by sumoylation (38) and TRIM28 was shown to have Sumo E3 ligase activity (3, 11, 39, 40, 41), we examined whether TRIM28 might regulate TFE3 stability through sumoylation. Yet, we detected no increase in TFE3 sumoylation by TRIM28 (Fig. S4*E*). Instead, we found TRIM28 increased the TFE3 ubiquitination level when TRIM28 was coexpressed with TFE3 (Fig. 4*D*). Consistently, knocking down TRIM28 decreased ubiquitination of endogenous TFE3 (Fig. 4*E*). The RING finger domain is a prototypical E3 ligase domain (42). To determine whether N-terminal RING finger domain in TRIM28 was necessary for the TFE3 ubiquitination, we mutated the conserved C65 and C68 to alanine in the TRIM28 N-terminal RING finger domain. Immunoprecipitation and Western blot results showed this mutant no longer promoted TFE3 ubiquitination (Fig. 4*F*). Consistently, this mutant showed diminished activity to decrease the TFE3 protein level and RCC cell proliferation (Figs. 4, *G*–*I* and S4*F*). These data showed TRIM28 promoted ubiquitin-proteasome-mediated degradation of TFE3 through its RING finger domain.

### TFE3 localizes to RCC cell nucleus

**Figure 5:**
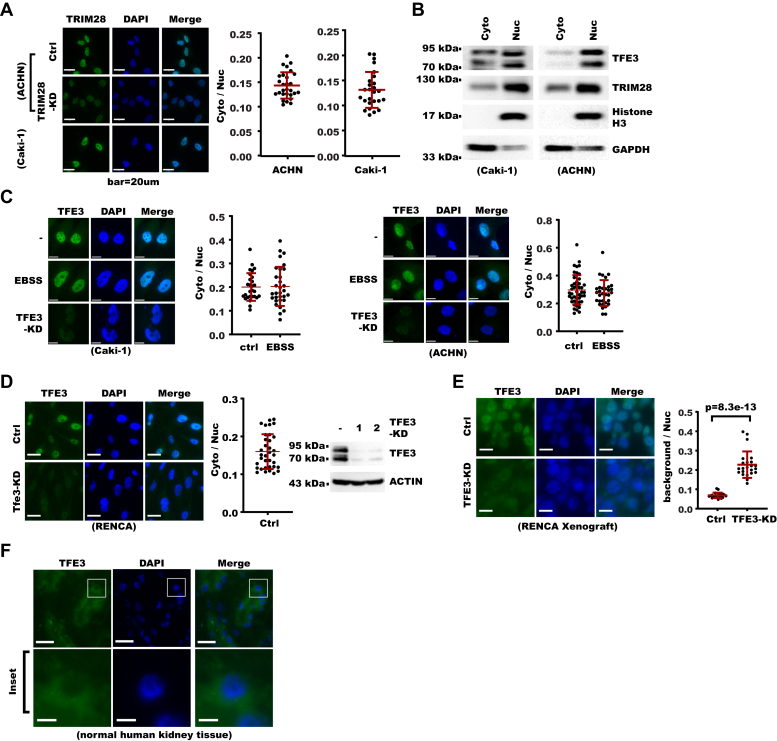
**TFE3 localizes to renal cell carcinoma cell nucleus.***A*, immunostaining for TRIM28 in control and TRIM28-KD cells (the scale bar represents 20 μm). Shown on the *left* are photos from fluorescent microscopy. Shown on the *right* is a summary of the cytoplasm/nucleus ratio of TRIM28 staining signal in each cell with mean and standard deviation shown in red horizontal bars (26 cells for Caki-1, 28 cells for ACHN). *B*, Caki-1 and ACHN cells were fractionated into cytoplasm and nuclear fractions. Fractions were analyzed with Western blot. GAPDH serves marker for cytoplasm while Histone H3 serves as marker for nuclei. *C*, cells were treated with Earle’s balanced salt solution (EBSS) for 4 h and then analyzed with immunostaining with nuclei counterstained with DAPI. TFE3-KD cells were included to show signal specificity. Shown on the *left* are photos from fluorescent microscopy (the scale bar represents 20 μm). Shown on the *right* is a summary of the cytoplasm/nucleus ratio of TFE3 staining signal in each cell with mean and standard deviation shown in *red horizontal bars* (for Caki-1: n = 28 cells for ctrl, 31 cells for EBSS; for ACHN: n = 45 cells for ctrl, 32 cells for EBSS). *D*, control or Tfe3-KD RENCA cells were analyzed with immunostaining and Western blot. Shown on the *left* are photos from fluorescent microscopy (the scale bar represents 20 μm). In the *middle* is a summary of the cytoplasm/nucleus ratio of Tfe3 staining signal in each cell with mean and standard deviation shown in *red horizontal bars* (n = 35 cells for Ctrl). Shown on the *right* are Western blot results. *E*, xenografts from control and Tfe3-KD RENCA cells were analyzed with immunostaining. The *left panel* shows photos from fluorescent microscopy (the scale bar represents 10 μm). On the *right* is a summary of the background/nucleus ratio of Tfe3 staining signal with mean and standard deviation shown in *red horizontal bars* (n = 20 cell for Ctrl, 23 cells for KD). *p* Value was calculated from Student's *t* test. *F*, immunostaining for TFE3 in noncancerous human kidney frozen section (the scale bar represents 20 μm). Inserts framed in white are further magnified at the *bottom* (the scale bar represents 5 μm).

Conventionally, TRIM28 is considered a nuclear protein while TFE3 mainly localizes to cytoplasm under normal conditions, so the significant effect of TRIM28 on TFE3 protein level seemed intriguing to us. We confirmed that both endogenous and exogenous TRIM28 localized to the nucleus with immunofluorescence and subcellular fractionation (Figs. 5, *A* and *B* and S5*A*). For immunofluorescence, diminished TRIM28 signal in TRIM28-KD cells indicated the specificity of the staining. Immunostaining on RCC samples in The Human Protein Atlas also supported TRIM28's major nuclear localization (Fig. S5*B*). Unexpectedly, fractionation and immunostaining experiments showed endogenous TFE3 also mainly localized to cell nucleus in RCC cells (Figs. 5, *B* and *C* and S5*C*). For immunofluorescence, diminished TFE3 signal in TFE3-KD cells indicated the specificity of the staining. Similarly, exogenous TFE3 also mainly localized to nuclei (Fig. S5*D*). Such localization pattern was also seen in cells treated with Earle’s balanced salt solution or TORIN1, which served as positive control for the immunostaining. Earle’s balanced salt solution rendered starvation while TORIN1 inhibited mTOR, both of which were known to promote nuclear localization of MITF family members (27). Immunostaining showed TFE3 also mainly localized to the nucleus in RENCA mouse kidney cancer cells and decreased TFE3 signal in TFE3-KD cells indicated specificity of the signal (Fig. 5*D*). We next further examined whether such localization pattern of TFE3 also occurred in kidney tumor samples. We first performed immunostaining on RENCA xenograft tumor sections generated from control or TFE3-KD RENCA cells. The result not only showed TFE3 mainly localized to the nucleus but also indicated the staining procedure was specific for TFE3 in tissue samples (Fig. 5*E*). With the same staining protocol, we then analyzed TFE3 localization in kidney, liver, and lung of normal BALB/c mice for comparison. We found TFE3 was generally evenly distributed in the kidney but preferentially distributed to the cytoplasm in liver and lung (Fig. S5*E*). For human RCC tissue, we analyzed the immunohistochemistry data of RCC patient samples from The Human Protein Atlas database. The result showed TFE3 had significant distribution to the nucleus in 11 of 12 samples, which was significantly higher than lung cancer tissues (Fig. S5*F*) and at least comparable with pancreatic cancer from the same database. For comparison, we performed immunostaining for TFE3 in kidney sections from five noncancer human donors who went through kidney needle aspirate examination. The result showed TFE3 mainly localized to the cytoplasm (Fig. 5*F*). In summary, these results showed TFE3 localized to the nucleus in RCC cells constitutively, unlike the noncancer kidney tissues.

### TFE3 promotes RCC cell autophagy and proliferation

**Figure 6:**
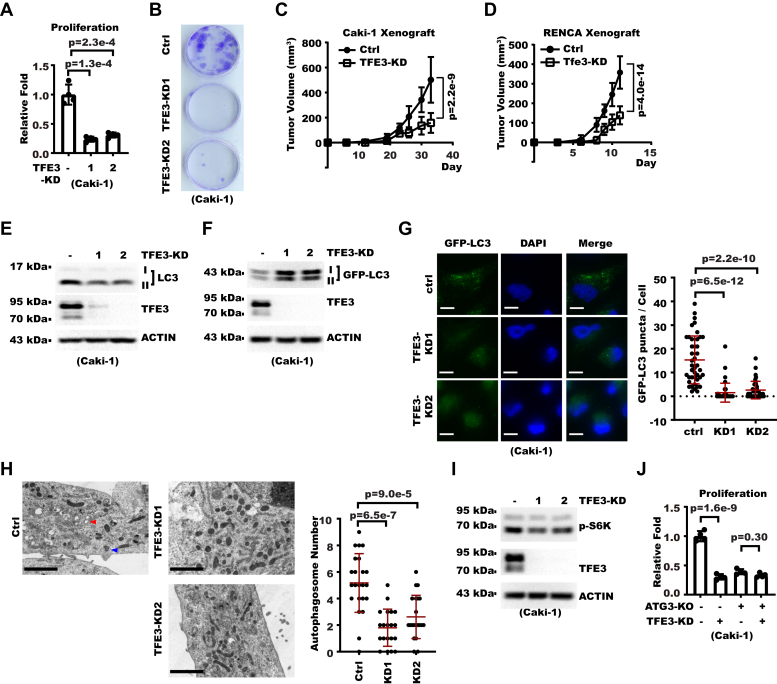
**TFE3 promotes renal cell carcinoma cell autophagy and proliferation.***A*, proliferation of control or TFE3-KD Caki-1 cells was analyzed by cell counting. Shown is relative proliferation fold over 7 days. Error bars denote standard deviation of four biological replicates. *p* Values were calculated from Student's *t* test. *B*, a total of 1000 control or TFE3-KD Caki-1 cells were seeded into 3.5-cm dishes. Cell colonies were stained with crystal violet 14 days later. *C*, a total of 5∗10∧6 control or TFE3-KD Caki-1 cells were inoculated subcutaneously to flanks of NOG mice. Shown are proliferation curve of xenograft tumors. Error bars denote standard deviation of five mice. *p* Value was calculated with two-way ANOVA. *D*, a total of 2∗10∧6 control or Tfe3-KD RENCA cells were inoculated subcutaneously to flanks of BALB/c mice. Shown are proliferation curves of xenograft tumors. Error bars denote standard deviation of seven mice. *p* Value was calculated with two-way ANOVA. *E*, TFE3 was knocked down in Caki-1 cells. Whole cell extracts (WCEs) were analyzed with Western blot (WB). *F*, TFE3 was knocked down in Caki-1 cells stably expressing GFP-LC3. WCEs were analyzed with WB. *G*, TFE3 was knocked down in Caki-1 cells stably expressing GFP-LC3. Cells were then analyzed with fluorescent microscopy with nuclei counterstained with DAPI. On the *left* are photos from fluorescent microscopy (the scale bar represents 10 μm). On the *right* is a summary of the GFP-LC3 puncta number in each cell with mean and standard deviation shown in *red horizontal bars* (n = 42 cells in ctrl, 40 cells in KD1, 38 cells in KD2). *p* Values were calculated from Student's *t* test. *H*, TFE3 was knocked down in Caki-1 cells. Cells were then analyzed with transmission electron microscopy. Shown on the *left* are TEM images (the scale bar represents 2 μm) where the *red arrowhead* denotes emptied lysosome and the *blue arrowhead* denotes autophagosome. Shown on the *right* is statistical analysis of autophagosome/autolysosome number in each cell with mean and standard deviation shown in *red horizontal bars* (23 cells in ctrl, 20 cells in KD1, 21 cells in KD2). *p* Values were calculated from Student's *t* test. *I*, TFE3 was knocked down in Caki-1 cells. WCEs were analyzed with WB. *J*, TFE3 was knocked down in control or ATG3-KO Caki-1 cells. Cell proliferation was examined by cell counting. Shown are relative folds of proliferation over 7 days. Error bars denote standard deviation of four biological replicates. *p* Values were calculated from one-way ANOVA with correction for multiple comparison.

We showed above TRIM28 could regulate autophagy and proliferation in RCC cells, so we next investigated the potential effect of TFE3 on these processes. TFE3-KD significantly decreased cell proliferation *in vitro* (Figs. 6, *A* and *B* and S6, *A* and *B*). To study whether a similar effect occurred in a mouse model, we inoculated Caki-1 cells to immunodeficient mice and found TFE3-KD inhibited tumor growth (Fig. 6*C*). In RENCA syngenic mouse tumor model, Tfe3-KD similarly decreased tumor growth (Fig. 6*D*). Consistently, a higher TFE3 protein level correlated with worse patient survival in patients with RCC from the CPTAC dataset (Fig. S6*C*). Next, we examined whether the effect of TFE3 on RCC cell proliferation was related to cell autophagy. We first examined the effect of TFE3 on previously described autophagy markers. TFE3-KD decreased lipidation of endogenous LC3 (Fig. 6*E*). In addition, TFE3-KD also decreased GFP-LC3 puncta number and inhibited GFP-LC3 degradation in RCC cell stably expressing GFP-LC3 (Figs. 6, *F* and *G* and S6*D*). To directly examine the effect of TFE3 on autophagosome and lysosomes, we then subjected TFE3-KD cells to TEM. The result confirmed TFE3-KD decreased the amount of autophagosome/autolysosome (Fig. 6*H*) and identified enlarged lysosomes filled with undigested cargos (Fig. S6*E*). Consistently, when we immunostained lysosome marker LAMP1, we confirmed TFE3-KD cells had increased lysosome size (Fig. S6*F*). These results all showed TFE3 was necessary for RCC cell autophagy. Consistently, our GSEA analysis also confirmed that TFE3 level positively correlated with autophagic gene expression in patients with RCC (Fig. S6*G*). Then we examined whether TFE3 regulates cell proliferation through autophagy. As we found earlier the effect of TRIM28 on mTOR activity, we analyzed TFE3-KD cells and found TFE3-KD decreased p-S6K, a marker of mTOR activity (Fig. 6*I*). To directly examine whether autophagy was necessary for TFE3-mediated cell proliferation, we then performed TFE3-KD in ATG3-KO cells. We found TFE3-KD exhibited little effect on cell proliferation in ATG3-KO cells (Fig. 6*J*). All these results from TFE3-KD were reminiscent of TRIM28-OE. Collectively, these data showed TFE3 promoted RCC cell proliferation and autophagy.

### TFE3 recruits KDM6A to autophagic gene promoters

**Figure 7:**
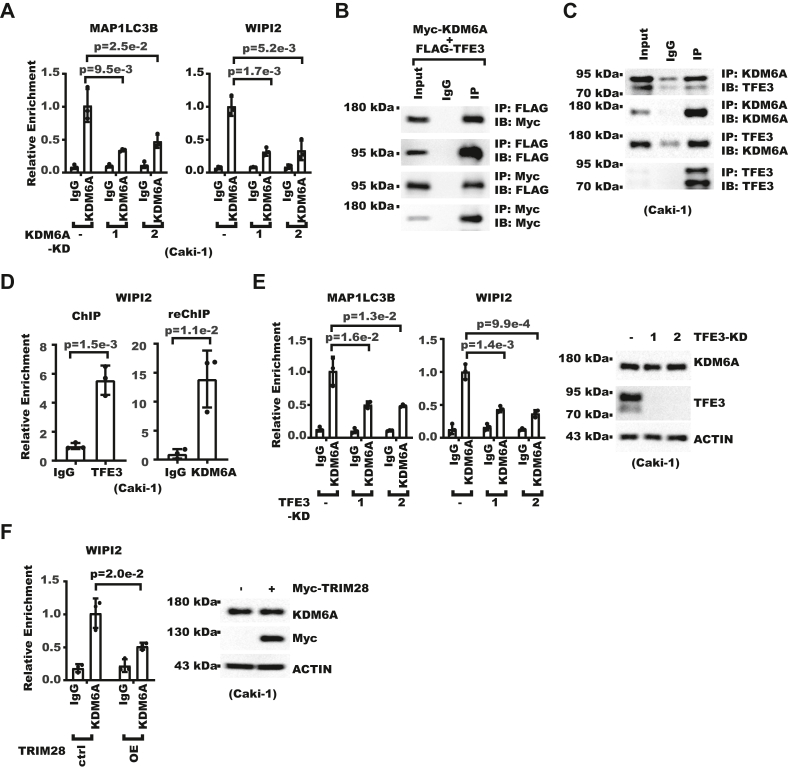
**TFE3 recruits KDM6A to autophagic gene promoters.***A*, KDM6A enrichment on indicated gene promoters was analyzed with chromatin immunoprecipitation (ChIP) followed by real-time PCR for control or KDM6A-KD Caki-1 cells. Error bars denote standard deviation of three technical replicates. *p* Values were calculated from Student's *t* test. *B*, Myc-KDM6A and FLAG-TFE3 were coexpressed in 293T cells. Cells were then lysed 48 h later for coimmunoprecipitation Western blot (WB) assay. Input denotes 1% input. *C*, interaction between endogenous KDM6A and TFE3 in Caki-1 cells was analyzed with coimmunoprecipitation WB assay. Input denotes 1% input. *D*, shown is the ChIP-reChIP result for the colocalization of TFE3 and KDM6A on WIPI2 promoter in Caki-1 cells. TFE3 antibody and KDM6A antibody were used in the first and second rounds of ChIP, respectively. Error bars denote standard deviation of three technical replicates. *p* Values were calculated from Student's *t* test. *E*, control or TFE3-KD cells were analyzed with ChIP and WB. On the *left*, KDM6A enrichment on indicated gene promoters was analyzed with ChIP followed by real-time PCR. Error bars denote standard deviation of three technical replicates. *p* Values were calculated from Student's *t* test. Shown on the *right* is WB for whole cell extracts. *F*, control or TRIM28-OE cells were analyzed with ChIP and WB. On the *left*, KDM6A enrichment on WIPI2 promoter was analyzed with ChIP followed by real-time PCR. Error bars denote standard deviation of three technical replicates, and *p* value was calculated from Student's *t* test. Shown on the *right* is WB for whole cell extracts.

Although change in chromatin status is widely considered a prerequisite for eukaryotic transcription regulation, little is known about how TFE3 affects chromatin status. We analyzed published ChIP-Seq data in human kidney cells and noticed TFE3 and KDM6A both localized to the promoter of target genes (Fig. S7*A*). Interestingly, KDM6A localized to many of the known TFE3 targets, with concordant H3K4me3, a marker for active promoters (Fig. S7*B*). We performed ChIP experiment and detected KDM6A enrichment on autophagic gene promoters (Fig. 7*A*). KDM6A ChIP signals could be decreased by KDM6A knockdown, supporting the specificity of ChIP signal (Fig. 7*A*). To examine the potential connection between TFE3 and KDM6A, we performed immunoprecipitation and Western blot and found both exogenous and endogenous TFE3 could interact with KDM6A (Fig. 7, *B* and *C*). We performed ChIP-reChIP and further confirmed colocalization of TFE3 and KDM6A to WIPI2 gene promoter (Fig. 7*D*). To examine whether TFE3 was required for KDM6A recruitment to autophagic gene promoters, we compared KDM6A ChIP signals in control and TFE3-KD cells. The result showed TFE3-KD decreased KDM6A enrichment without changing the KDM6A protein level (Fig. 7*E*). As TRIM28 decreased TFE3 level, we reasoned it might decrease KDM6A enrichment on autophagic genes as well. ChIP analysis confirmed TRIM28 overexpression indeed decreased KDM6A enrichment on WIPI2 gene promoter (Fig. 7*F*). Collectively, these results showed KDM6A could be recruited by TFE3 to autophagic gene promoters.

### KDM6A promotes autophagic gene expression through H3K4 methylation

**Figure 8:**
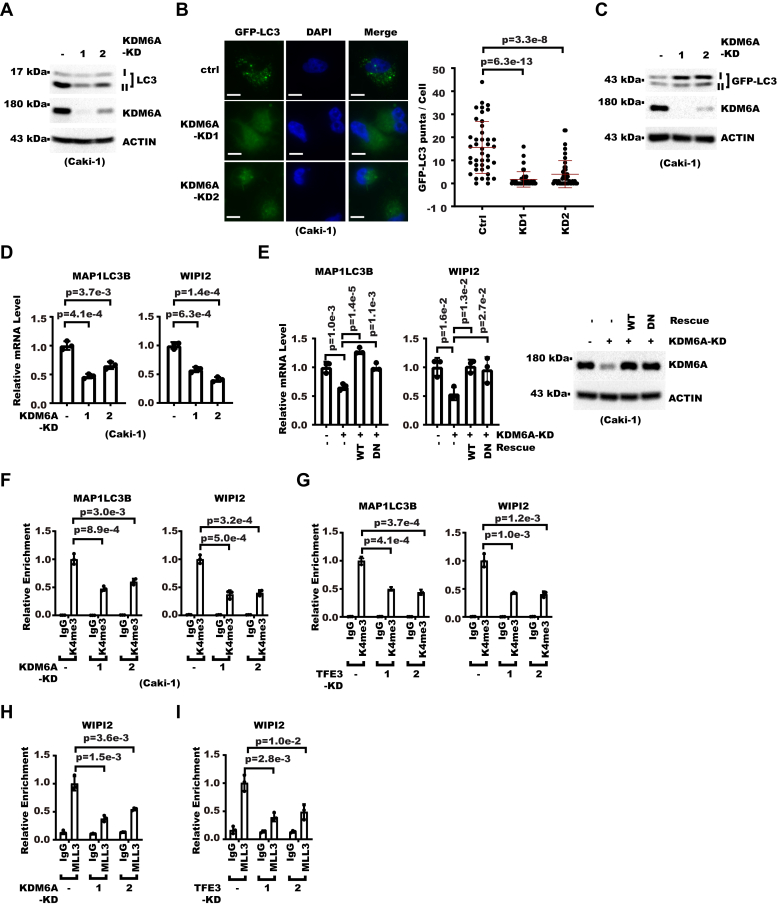
**KDM6A promotes autophagic gene expression through H3K4 methylation.***A*, whole cell extracts of control or KDM6A-KD Caki-1 cells were analyzed with Western blot (WB). *B*, KDM6A was knocked down in Caki-1 cells stably expressing GFP-LC3. Shown on the *left* are photos from fluorescent microscopy with nuclei counterstained with DAPI (the scale bar represents 10 μm). Shown on the *right* is statistical analysis for GFP-LC3 puncta number in each cell with mean and standard deviation shown in *red horizontal bars* (n = 42 in ctrl, 44 in KD1, 45 in KD2). *p* Values were calculated from Student's *t* test. *C*, KDM6A was knocked down in Caki-1 cells stably expressing GFP-LC3. Whole cell extracts were then analyzed with WB. *D*, control or KDM6A-KD Caki-1 cells were analyzed with real-time RT-PCR. Shown are relative mRNA levels of indicated genes. Error bars denote standard deviation of three technical replicates. *p* Values were calculated from Student's *t* test. *E*, KDM6A was knocked down in Caki-1 cells and then KDM6A wildtype (WT) or inactive mutant (DN) was rescue-expressed. Cells were analyzed with real-time RT-PCR or WB. Shown on the *left* are relative mRNA levels of indicated genes as measured by real-time RT-PCR. Error bars denote standard deviation of three technical replicates. *p* Values were calculated from one-way ANOVA with correction for multiple comparison. Shown on the *right* are WB results. *F* and *G*, H3K4me3 enrichment on indicated genes was analyzed with chromatin immunoprecipitation followed with real-time PCR in control and KDM6A-KD (*F*) or TFE3-KD (*G*) Caki-1 cells. Error bars denote standard deviation of three technical replicates. *p* Values were calculated from Student's *t* test. *H* and *I*, MLL3 enrichment on indicated genes was analyzed with chromatin immunoprecipitation followed with real-time PCR in control and KDM6A-KD (*H*) or TFE3-KD (*I*) Caki-1 cells. Error bars denote standard deviation of three technical replicates. *p* Values were calculated from Student's *t* test.

We showed above KDM6A was recruited to autophagic gene promoters by TFE3. We next examined whether KDM6A contributed to RCC cell autophagy. Western blot showed KDM6A-KD decreased endogenous LC3 lipidation (Fig. 8*A*). Consistently, KDM6A-KD decreased GFP-LC3 puncta number and inhibited GFP-LC3 degradation (Figs. 8, *B* and *C* and S8*A*) in RCC cells stably expressing GFP-LC3. These results showed KDM6A-KD was required for RCC cell autophagy. As our earlier data showed autophagy was critical for RCC cell proliferation, we next determined whether KDM6A also contributed to RCC cell proliferation. We found KDM6A knockdown indeed decreased RCC cell proliferation (Fig. S8*B*). We next examined whether KDM6A also regulated autophagic gene expression like TFE3. Indeed, KDM6A-KD decreased the expression of autophagic genes (Figs. 8*D* and S8*C*). Consistently, our GSEA analysis confirmed KDM6A positively correlated with autophagic gene expression among patients with RCC whose TFE3 level was relatively higher than TFEB and MITF (Fig. S8*D*). KDM6A has intrinsic H3K27me3 demethylase activity. To determine whether this activity was required for regulation of autophagic gene expression, we rescue-expressed KDM6A-WT or inactive H1146A mutant (DN) in KDM6A-KD cells. The result showed not only KDM6A-WT but also KDM6A-DN significantly restored expression of autophagic genes, indicating KDM6A demethylase activity was not necessary (Fig. 8*E*). In addition to demethylating H3K27me3, KDM6A was known to promote gene activation together with MLL3/4 through H3K4me3 (31, 32, 33). So we examined whether KDM6A affected the H3K4me3 level on autophagic gene promoters and found KDM6A-KD decreased H3K4me3 (Fig. 8*F*). Consistently, TFE3-KD also decreased H3K4me3 but not H3K27me3 on autophagic gene promoters (Figs. 8*G* and S8*E*). Analysis of published ChIP-Seq result on RCC patients samples also confirmed that H3K4me3 but not H3K27me3 was enriched at autophagic gene loci (Fig. S8*F*). Consistent with the decrease in H3K4me3, KDM6A-KD as well as TFE3-KD decreased the enrichment of MLL3 on the autophagic gene promoters (Figs. 8, *H* and *I* and S8*G*). In summary, these data showed KDM6A promoted autophagic gene expression through H3K4 methylation.

## Discussion

### TRIM28–TFE3–KDM6A axis regulates RCC cell proliferation and tumor growth

**Figure 9:**
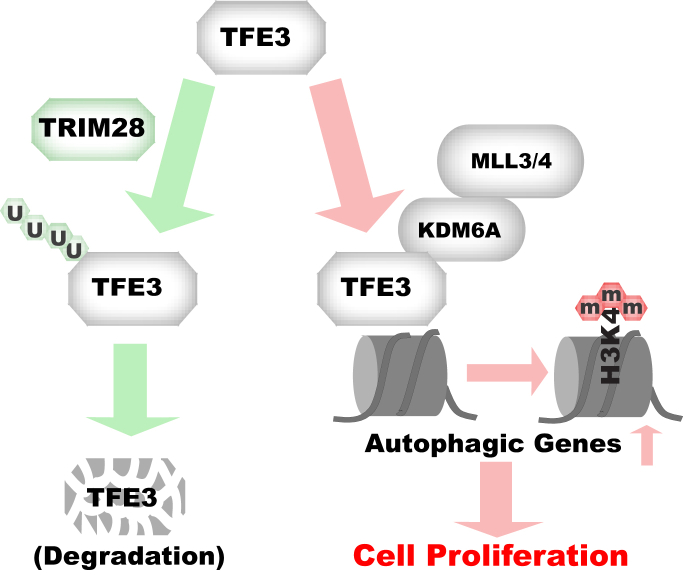
**Working model.** In renal cell carcinoma cells, TRIM28 could promote ubiquitination and degradation of TFE3, leading to decreased autophagy and cell proliferation. Otherwise, TFE3 accumulates in the cell nucleus, where it interacts with and recruits KDM6A to autophagic genes to increase local H3K4me3 and gene expression, leading to increased autophagy and cell proliferation in renal cell carcinoma.

TRIM28 has multiple biochemical activities. While TRIM28 loss of function was seen in Wilms kidney blastoma, little is known about the function and the underlying mechanism of TRIM28 in etiology of kidney malignancy. On the other hand, as a pivotal transcription factor for autophagy-lysosome genes, TFE3 activation due to gene fusion also notably causes kidney malignancy. However, it is unclear whether and how TRIM28 and TFE3 are related in RCC etiology. In this study, we find higher TRIM28 level correlates with better RCC patient survival and TRIM28 suppresses RCC cell proliferation and tumor growth. Mechanistically, we show TRIM28 promotes ubiquitination and proteasome-mediated degradation of TFE3. Interestingly, we uncover TFE3 is constitutively localized to cell nucleus in RCC, which promotes RCC proliferation. Our study reveals a hitherto unappreciated link between TRIM28 and TFE3, two genes pivotal in etiology of kidney malignancy. In addition, we uncover a mechanism by which TFE3 regulates chromatin status at target genes. We find TFE3 functionally interacts with and recruits KDM6A to autophagic genes, which promotes the local H3K4me3 level. In summary, our study establishes a TRIM28–TFE3–KDM6A signal axis pivotal for RCC cell proliferation (Fig. 9).

### TRIM28 inhibits autophagy of RCC through TFE3

In tissues other than kidney, the impact of TRIM28 on autophagy was reported previously and attributed to different mechanisms. TRIM28 was reported to inhibit autophagy through promoting degradation of AMPK-α1 in lung, breast, and colon cancer cells (13), which depends on MAGE family proteins as adaptors (13). In addition, TRIM28, upon Histone deacetylase inhibitor treatment, partially translocates to the cytoplasm in MCF7 breast cancer cell, where it may promote autophagy through sumoylating and activating VPS34 (43). Here, we show TRIM28, which is localized to the nucleus of RCC cells, plays a pivotal role in RCC autophagy regulation through downregulating TFE3. We find that TRIM28 decreases LC3 lipidation, number of LC3 puncta, and autophagosomes with accompanying enlarged lysosomes, which all indicate defected autophagy-lysosome function.

Interestingly, in this study, we find an increased portion of TFE3 is localized to the RCC cell nucleus in contrast to normal tissue. Consistent with its localization, we find TFE3 is necessary for autophagy and cell proliferation in RCC. As MITF family proteins play critical roles in autophagy regulation, extensive study has been dedicated to regulation of TFEB and showed TFEB is subject to ubiquitination (44, 45). Yet, recent studies and ours show TFE3 also plays a significant role in cell autophagy (28, 46). Yet, little is known how TFE3 is regulated. As genetic evidence points to a critical role of TFE3 in kidney malignancy, we focused on RCC in this study. We have identified TRIM28 as a key regulator of TFE3 protein stability in RCC.

### KDM6A is recruited by TFE3 for autophagic gene activation

It is widely held that eukaryotic transcription has to engage chromatin change, yet it remains elusive how TFE3 affects local chromatin modification (47). In this study, we find that TFE3 can recruit KDM6A to autophagic gene promoters and increase local H3K4me3. Consistently, KDM6A is also necessary for autophagy gene expression and autophagy in RCC cells. We find this function of KDM6A is independent of its H3K27me3 demethylase activity. Instead, KDM6A achieves this function through engaging MLL3/4 and promoting H3K4 methylation, another modality of KDM6A function identified previously (31, 32, 33). Interestingly, in drosophila, KDM6A was also previously shown to regulate autophagy gene expression during salivary gland development through H3K27 demethylation (48), indicating KDM6A may have an evolutionarily conserved role in autophagy regulation albeit through different mechanisms. Pertinently, JMJD3, a homologue of KDM6A, can also target and increase the expression of autophagic genes (49). Once considered a cytoplasmic event, it is now established that autophagy is also regulated by gene expression (24). Histone modifications function at the interface of environmental changes and gene expression, dysregulation of which contributes to human diseases including cancer. Here, our study and previous studies altogether show dysregulation of histone modification makes significant contribution to cancer etiology through autophagy.

## Experimental procedures

### Cell culture

Caki-1 cells were cultured in McCoy's 5A (Procell #PM150710). ACHN cells were cultured in minimum essential medium supplemented with nonessential amino acids (Procell #PM150410). 786-O cells were cultured in RPMI-1640 (Procell #PM150110). HEK-293T cells were cultured in Dulbecco’s modified Eagle’s medium (Procell #PM150210). All media were supplemented with 10% fetal bovine serum. RENCA cells were cultured in RPMI-1640-based complete medium (Procell #CM-0568). All cells were cultured in 5% CO2 incubator. Cycloheximide (Sigma, #C7698), MG132 (MCE, #HY-13259), and TORIN1 (MCE, #HY-13003) were added to cell culture at a final concentration of 25 mg/ml, 25 μM, and 1 μM, respectively, where indicated.

### Stable GFP-LC3 cell lines

Caki-1 cells were transduced with lentivirus expressing GFP-LC3B (human). After selection with 10 μg/ml blasticidin for 1 week, single cells were seeded into 96-well plate, further propagated, and validated.

### Cell proliferation assay

Cell proliferation assay was performed as we reported (50). Briefly, the same amount of cells were seeded into 3.5-cm dish, and cell number was counted 7 or 9 days later as specified. The fold of proliferation was calculated as the ratio between cell number in the end and the cell number at the beginning. Fold of proliferation was then normalized to the control group to get the “relative proliferation fold.”

### Colony formation assay

Colony formation assay was performed as we reported (51). Briefly, 1000 Caki-1 or ACHN cells were seeded into 3.5-cm dishes. The medium was refreshed every week. After 2 weeks, cells were fixed with methanol and stained with 0.5% crystal violet.

### Xenograft experiment

All animal experiments were performed following the institute guidelines and approved by Ethics committee of Tongji Medical College. The mice were acclimated to the new environment for at least 1 week. Mice were housed in ventilated cages in a temperature-controlled room (21 °C ± 1 deg. C) with a 12 h light/12 h darkness cycle. Food and water were available *ad libitum*. Mice were randomly assigned to experimental groups. A total of 5∗10∧6 Caki-1 cells were resuspended in a 1:1 mixture of PBS and Matrigel (BD #354248) and then inoculated subcutaneously into flanks of five 6-week-old male NOG mice (Charles River Beijing). A total of 2∗10∧6 RENCA control or TFE3-KD cells were inoculated subcutaneously into flanks of seven 6-week-old female BALB/c mice (Charles River Beijing). Tumor growth was measured with caliper every 3 to 7 days. Tumor volume was estimated by the formula 0.5∗L∗W∗W (L mean long diameter, W means short diameter). Mice were sacrificed before the estimated volume of any tumor reaches 1 cm^3^.

### Western blot

Western blot was performed exactly as we described in detail (50). Briefly, protein samples were first separated by SDS-PAGE and then transferred to PVDF membrane with a Mini Trans-Blot Module (Bio-Rad). Membranes were then blocked and incubated with primary antibodies and secondary antibodies sequentially. Antibodies used are anti-LC3A/B (CST #4108), anti-P62 (CST #5114), anti-TRIM28 (Zen Bio #200280), anti-GFP (ProteinGene #2057), p-S6K (CST #9234), ATG3 (CST #3415), TFE3 (CST #14779), TFEB (CST #4240), MITF (CST #97800), KDM6A (CST #33510), p-S6K(CST #9234), ATG3 (CST #3415), AMPKα (CST #5831), Histone H3 (Abcam #ab1791), GAPDH (ABclonal #AC033), β-ACTIN (ABclonal #AC026), Ubiquitin (Santa Cruz #sc-8017), Myc Tag (Proteintech #16286-1-AP; Santa Cruz #sc-40), FLAG tag (Sigma #A8592), HA-tag (Covance #MMS-101P; CST #3724), and GST tag (Proteintech #HRP-66001).

### Immunofluorescence

Use of leftover frozen sections produced from fine-needle aspirate of noncancer donors was approved by the Ethics Committee of Tongji Medical College and abides by the Declaration of Helsinki principles. Patients went through fine-needle aspiration for pathology diagnosis of nephrotic syndrome (one patient, 7-year-old male), lupus nephritis (two patients, 12-year old female), Henoch–Schonlein purpura (8-year-old male and 14-year-old female). For immunofluorescence on human tissue, frozen sections generated from needle aspirate of noncancer donors were first fixed with 4% paraformaldehyde for 15 min. Slides were then washed and permeabilized with 0.5% Triton-X100 in cold PBS. Slides were then blocked with 1% bovine serum albumin for 1 h, incubated with primary antibodies for 1 h, incubated with secondary antibodies for 1 h. Nuclei were counterstained with 1.5 μM DAPI (4′,6-diamidino-2-phenylindole) for 5 min. Slides were then washed three times with PBST (PBS + 0.2% Tween-20) and mounted onto antifading mounting medium (Abcam AB104135). Immunofluorescence for mouse tissue was performed similarly except mice kidneys or xenograft tumors were first fixed with 4% paraformaldehyde overnight. Immunofluorescence for adherent cell culture was performed with the same procedure as for frozen section. Antibodies used for immunostaining are TFE3 (CST #14779), TRIM28 (ZenBio #200280), and LAMP1 (CST #15665).

### Transmission electron microscopy

The cell culture medium was discarded and cells were fixed with 2.5 glutaraldehyde in PBS (Servicebio #G1102) at 4 °C for 2 to 4 h. Cell were then scraped and centrifuged to a pellet. Further sample processing was done at Servicebio Inc. Briefly, cell pellets were embedded in 1% agarose (water), washed three times with PBS (pH 7.4) for 15 min each time, and then fixed with 1% osmium tetroxide. Samples were then dehydrated in increasing concentrations of ethanol and acetone. Afterward, samples were embedded in SPI 812. Finally, 60- to 80-nm ultrathin sections were cut with Leica EM UC7 and stained with uranyl acetate and lead citrate. Ultrathin sections were examined with a transmission electron microscope (Hitachi HT7800) at 8000-fold magnification.

### Real-time RT-PCR

Sybgreen1-based real-time PCR was performed exactly as we described in detail on a Bio-Rad CFX connect PCR machine (50). Primers used are listed in Table S1.

### Chromatin Immunoprecipitation and reChIP

ChIP was performed exactly as we described (50). Briefly, cells were fixed with formaldehyde and chromatin was then sonicated into fragments. The cell lysate was then incubated with primary antibodies. Chromatin was captured with Protein A/G magnetic beads. DNA was then purified for real-time PCR analysis. Primers used are listed in Table S1. Antibodies used for ChIP are anti-H3K4me3 (Abcam #AB8580), H3K27me3 (Abcam #AB6002), Histone H3(Abcam #ab1791), TFE3 (Proteintech #14480-1-AP), KDM6A (CST #33510), MLL3 (Proteintech #28437-1-AP), and Rabbit IgG (Proteintech #B900610).

ChIP-reChIP was performed as we described (52). TFE3 antibody-bound chromatin was eluted with reChIP elution buffer (50 mM Tris-Cl pH 8.0, 10 mM DTT, 1% SDS) and diluted 10-fold with dilution buffer (50 mM Tris-Cl pH 8.0, 167 mM NaCl, 2.2 mM EDTA, 1.1% Triton X-100). KDM6A (CST #33510) antibody was then added for reChIP. Finally, DNA was purified and analyzed with real-time PCR. Primers used are listed in Table S1.

### Gene knockdown

ShRNA-based gene knockdown was performed exactly as we described (50). Synthesized DNA oligos corresponding to target sequences were annealed and ligated into lentivirus-based pLKO vector. Lentivirus was then produced by cotransfecting pLKO with psPAX2 and pMD2.G. The used oligo sequences are listed in Table S1.

### Gene knockout

Gene knockout was performed with the CRISPR-Cas9 system exactly as we described (50). Synthesized DNA oligos corresponding to guide RNA were annealed and ligated into lentivirus-based pLenti-CRISPR-V2 vector. Lentivirus was then produced by cotransfecting pLenti-CRISPR-V2 with psPAX2 and pMD2.G. The used oligo sequences corresponding to guide RNAs are listed in Table S1.

### Overexpression or rescue expression with lentivirus vector

Lentivirus-mediated gene expression was performed as we described (50). Expression cassettes were cloned into pLenti-EF1a vector carrying blasticidin resistance gene. Lentivirus was packaged to transduce target cells. Cells were then selected with blasticidin (10 μg/ml) for 1 week. ShRNA resistance was achieved by introducing synonymous mutation into shRNA-targeted sequence, and primers used are included in Table S1.

### *In vivo* ubiquitination assay

*In vivo* ubiquitination assay was performed exactly as we described (50). Cells were treated with 10 μM MG132 4 h before collection. Cells were then lysed in IPE150 buffer (20 mM Hepes pH 7.5, 150 mM NaCl, 0.5% NP40, 10% Glycerol) supplemented with 10 μM MG132 and 10 mM *N*-ethylmaleimide (Aladdin, #E100553). Exogenous or endogenous TFE3 was then immunoprecipitated with FLAG antibody (Sigma #A2220) or TFE3 antibody (Proteintech #14480-1-AP), respectively. The captured material was washed with IPE150 three times and analyzed with Western blot.

### *In vivo* sumoylation assay

Equal amounts of FLAG-TFE3, HA-SUMO, and Myc-TRIM28 plasmids were cotransfected into 293T cells. Forty-eight hours later, cells were lysed in IPE150 buffer (20 mM Hepes pH 7.5, 150 mM NaCl, 0.5% NP40, 10% Glycerol) containing 10 mM *N*-ethylmaleimide (Aladdin, #E100553). After sonication and centrifugation, the cell lysate was incubated with anti-FLAG M2-conjugated agarose beads for 4 h. After washing with IPE1000 (20 mM Hepes pH 7.5, 1000 mM NaCl, 0.5% NP40, 10% Glycerol) three times for 5 min each time, beads-captured material was analyzed with Western blot.

### Coimmunoprecipitation assay

Coimmunoprecipitation was performed as we described (50). Briefly, cells were lysed in IPE150 buffer with sonication. The cell lysate was cleared by centrifugation, and the supernatant was incubated with antibodies and immunoprecipitated with Protein A/G-conjugated agarose beads. Alternatively, the cell lysate was incubated with antibody-conjugated beads. Antibodies or antibody-conjugated beads used are TFE3 (Proteintech #14480-1-AP), KDM6A (CST #33510), TRIM28 (ZenBio #200280), Myc-Tag (Santa Cruz #sc-40), anti-FLAG-conjugated beads (Sigma #A2220). Immunoprecipitated material was analyzed with Western blot.

### Densitometry analysis of Western blot result

Densitometry analysis for Western blot results was performed with FIJI as we described (50). Western blot pictures were turned into 8-bit Gy, background subtracted, and bands were manually circled out and densitometry was measured with the “measure” function in FIJI. Density from each sample was then normalized to density of β-ACTIN from the same sample to get the relative level of each band. Afterward, the relative level from each sample was normalized to that of the control group.

### Analysis of ChIP-Seq data from GEO

Input and HA-TFE3 ChIP-Seq data for HK-2 cells were from GSE135490. KDM6A and H3K4me3 ChIP-Seq data for HEK293 were from GSE172141. H3K4me3 and H3K27me3 ChIP-Seq data of patients with kidney cancer were from GSE75597. Raw reads were first filtered with Trim-Galore and then aligned with bowtie2 (53) to human hg38 genome assembly. Alignments with MAPQ >30 were kept and genomic coverage in bigwig format (bin = 25 bp) was generated with Deeptools BamCoverage (54). Reads coverage at WIPI2 was viewed with UCSC genome browser or Integrative Genomics Viewer. Read coverage around a metagene (Transcription start site TSS to Transcription end site TES resized to 10 kB artificially) was calculated by Deeptools ComputeMatrix (bin = 25 bp) and then the column means of the matrix were used to make the profile plot with R.

### Survival analysis and Kaplan–Meier plot

Gene expression data and survival information of TCGA patients were downloaded from Genomic Data Commons data portal. Protein expression data for CPTAC patients with kidney cancer were downloaded from the CPTAC data portal. Patients were divided into two groups according to expression level of corresponding genes. Survival analysis and Kaplan–Meier plot were performed with R “survival” package as we described (51).

### Analysis of GFP-LC3 or LAMP1 puncta

Photos were taken with QImaging Retiga R6 Monochrome camera connected to Zeiss-A1 Axiovert A1 fluorescence microscopy equipped with 63× oil object lens. Exposure time for photos from the same batch of experiment was set at the same value for comparison. Photos were then processed and analyzed with FIJI software package. Briefly, photos were background-subtracted, made into binary with same threshold, processed with “watershed” tool in FIJI. GFP-LC3 particles were called with “analyze particle” function in FIJI with parameters: size 0.20 to 6.50 μm^2^, circularity 0 to 1. LAMP1 particles were called with “analyze particle” function in FIJI with parameters: size 0.066 to 6.50 μm^2^, circularity 0 to 1. Peculiar dots outside of cells were manually filtered out. Student's *t* test was used to statistically analyze the difference between particle sizes in different groups.

### GSEA analysis

GSEA analysis with preranked genes was performed with R package “fgsea,” and the result was validated with the GSEA application (version 4.2.1). Plot for GSEA was generated with R package “gggsea.” Genes were first ranked according to the Pearson correlation coefficient between log2 expression values of themselves and TFE3, TRIM28, or KDM6A where indicated. Autophagy gene set was adopted from previous publication (28). To sort out tumors whose TFE3 level was relatively higher than TFEB and MITF, RNA-Seq raw read counts from STAR were downloaded from the Genomic Data Commons data portal and then normalized with DESeq2. “TFE3 relatively higher” was defined as normalized counts of TFE3 higher than sum of TFEB and MITF multiplied with a factor 1.5, 2.0, or 3.0 as specified.

### Statistical analysis

For the xenograft tumor growth curve, *p* values were calculated with two-way ANOVA with Geisser–Greenhouse correction and *p* values for the interaction between time and group were shown in the graph. For GSEA, *p* values were obtained by permuting the gene set 1000 times. *p* Values for immunohistochemistry data from The Human Protein Atlas was calculated by Fisher exact test. *p* Values for protein stability assay were calculated by two-way ANOVA. *p* Values for all other assays were calculated by either Student's *t* test or one-way ANOVA as specified in the text. For one-way ANOVA, Sidak statistical hypothesis testing was used to correct for multiple comparison as recommended by GraphPad. All *p* values larger than 1e-15 were calculated with GraphPad 8.0. *p* Values less than 1e-15 were calculated with R 4.2.1. *p* Values (or *p*-adjusted for multiple comparison) less than 0.05 were deemed significant. All error bars denote standard deviation.

## Data availability

The data that support the findings of this study are available from the corresponding authors upon reasonable request.

## Supporting information

This article contains supporting information.

## Conflict of interest

The authors declare that they have no conflicts of interest with the contents of this article.
