# Supplementary figures and images for: TRIM28 represses renal cell carcinoma cell proliferation by inhibiting TFE3/KDM6A-regulated autophagy
Source: J Biol Chem. 2023 Mar 18;299(5):104621. doi: 10.1016/j.jbc.2023.104621 (PMC10141522; doi:10.1016/j.jbc.2023.104621)

# Figure S01

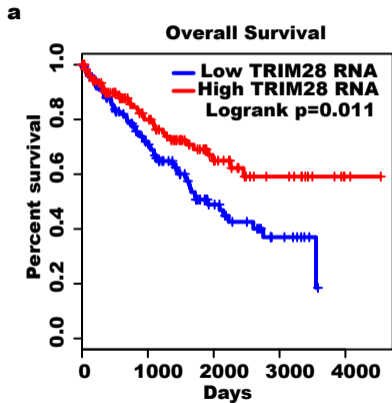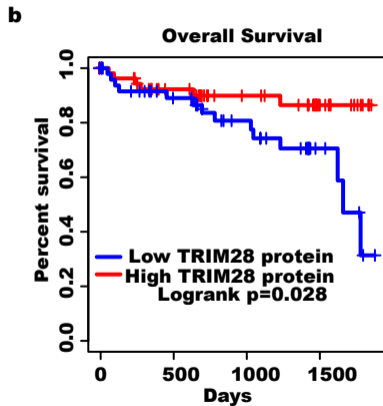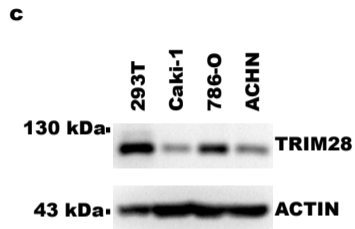

Supplement: Supporting Figure S1 [file mmc2.pdf]

**Figure S02**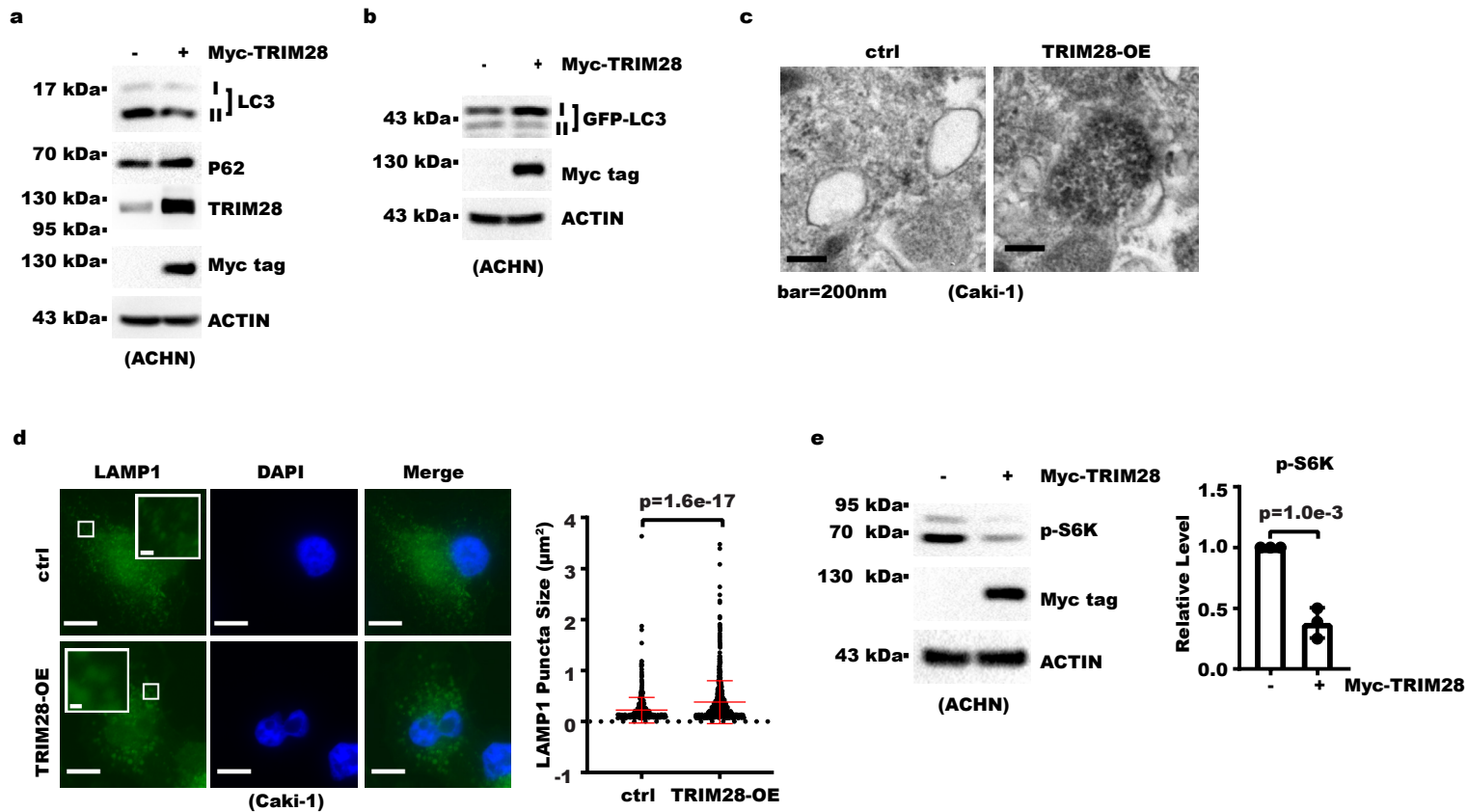

Supplement: Supporting Figure S2 [file mmc3.pdf]

**Figure S03**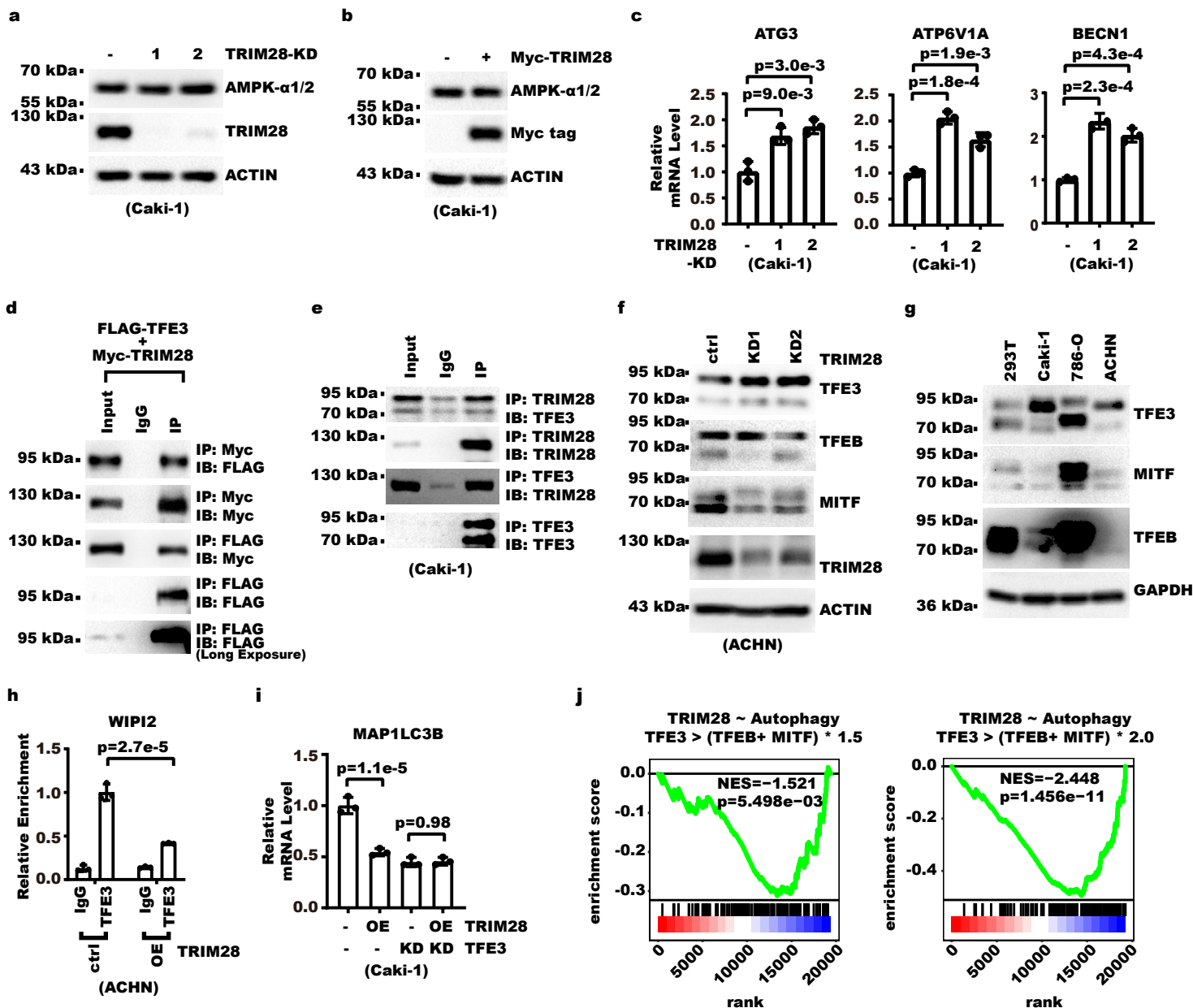

Supplement: Supporting Figure S3 [file mmc4.pdf]

**Figure S04**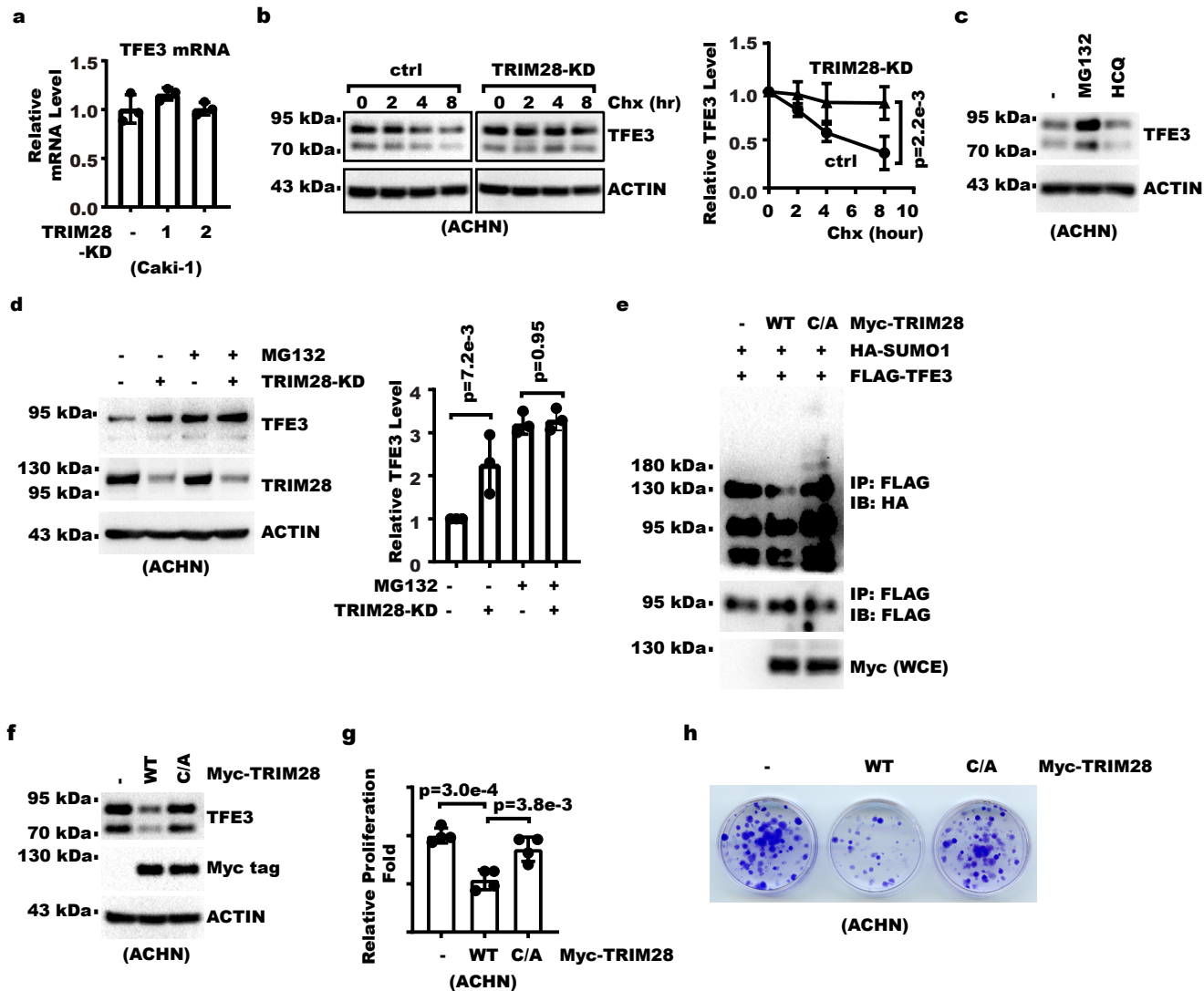

Supplement: Supporting Figure S4 [file mmc5.pdf]

**Figure S05**

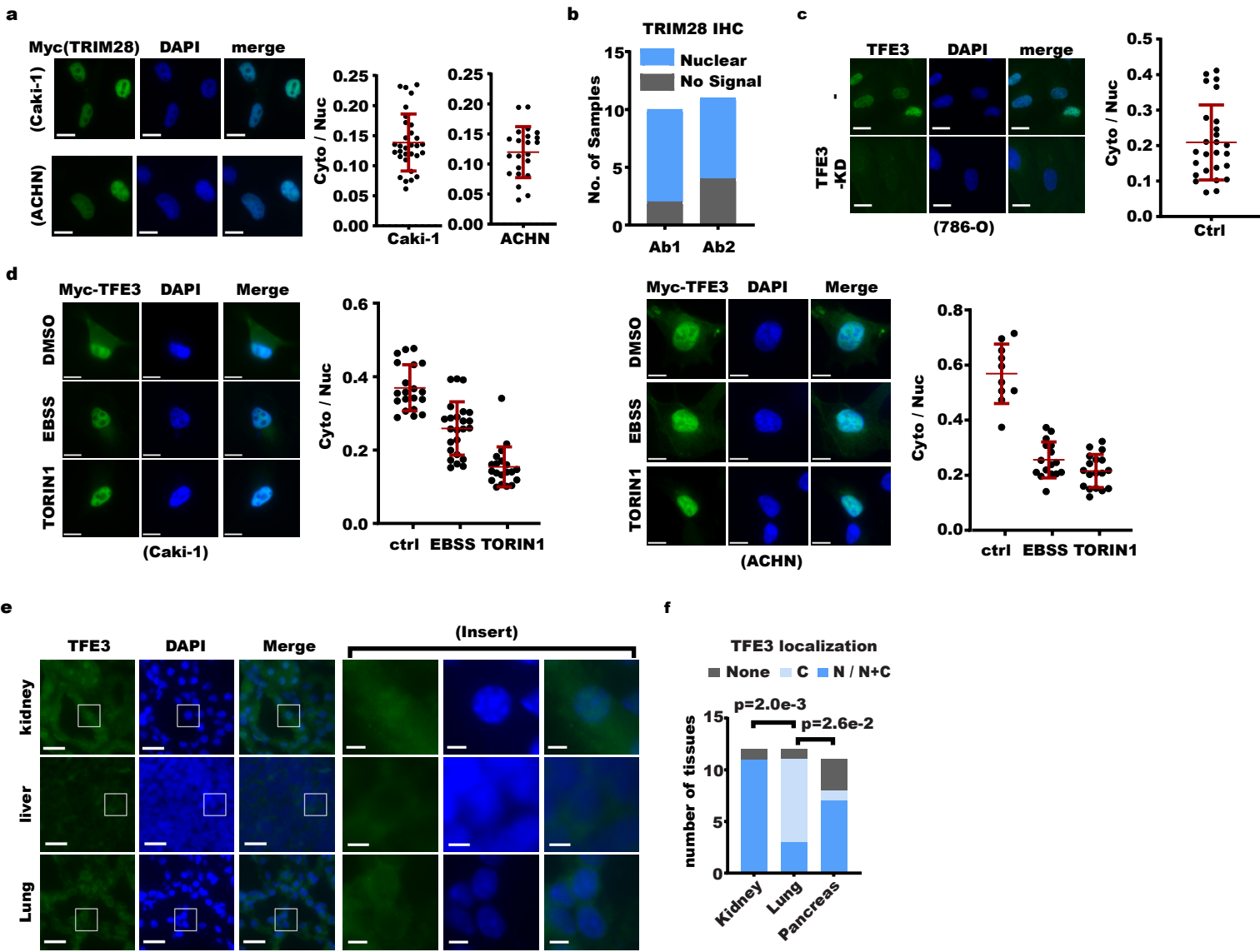

Supplement: Supporting Figure S5 [file mmc6.pdf]

**Figure S06**

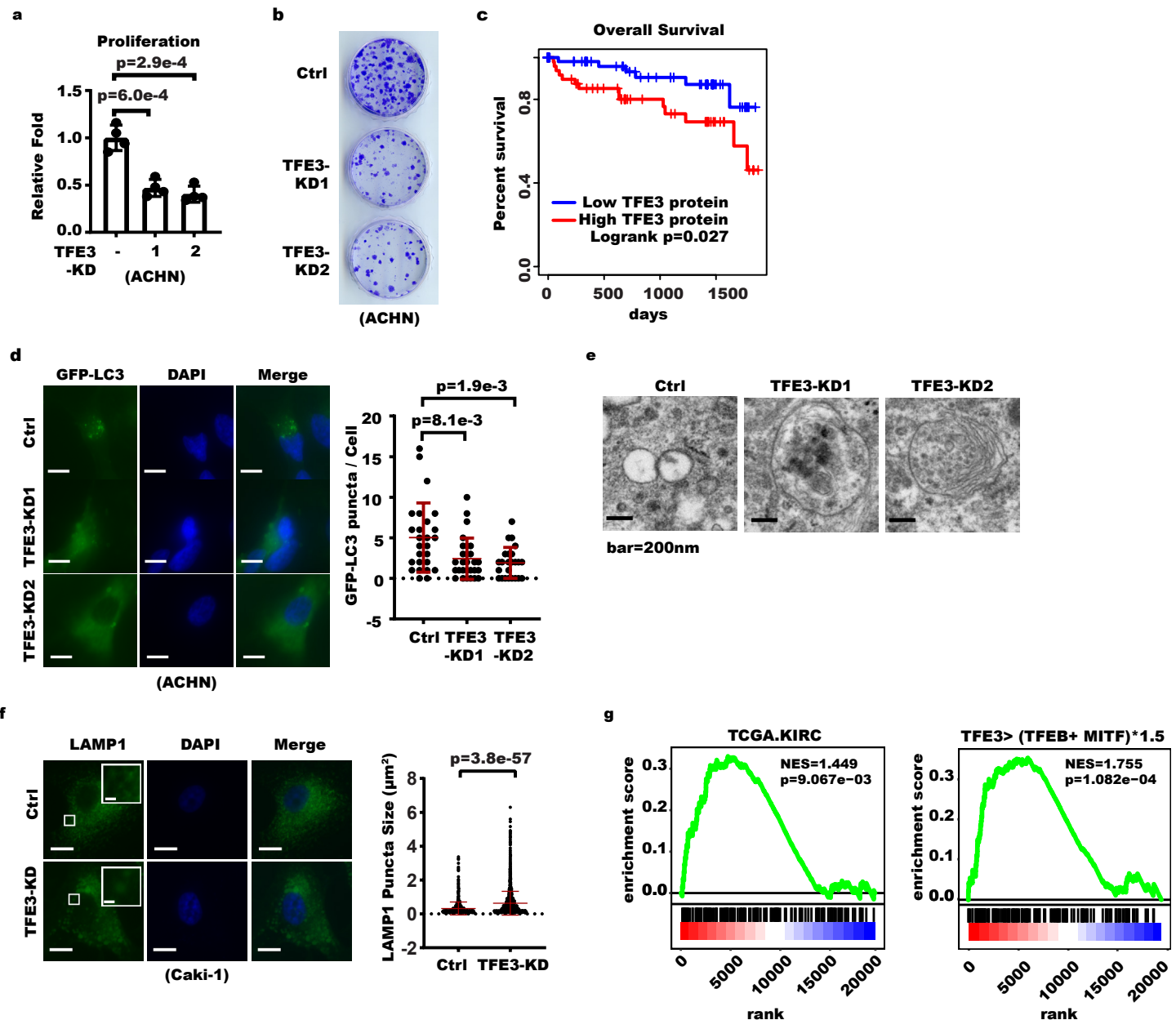

Supplement: Supporting Figure S6 [file mmc7.pdf]

# Figure S07

a

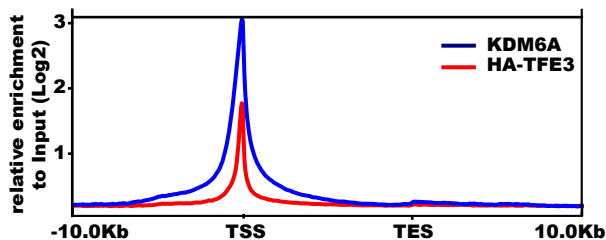

b

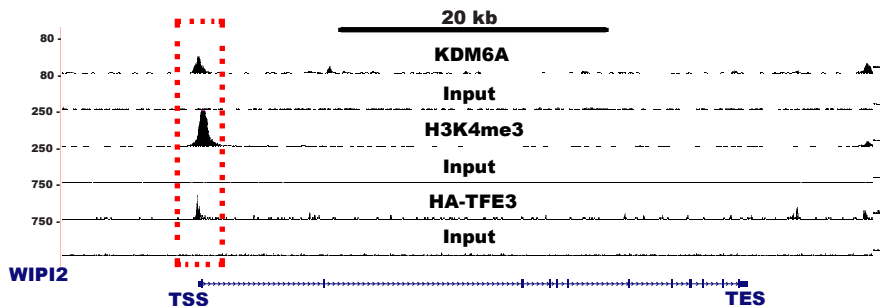

Supplement: Supporting Figure S7 [file mmc8.pdf]

**Figure S08**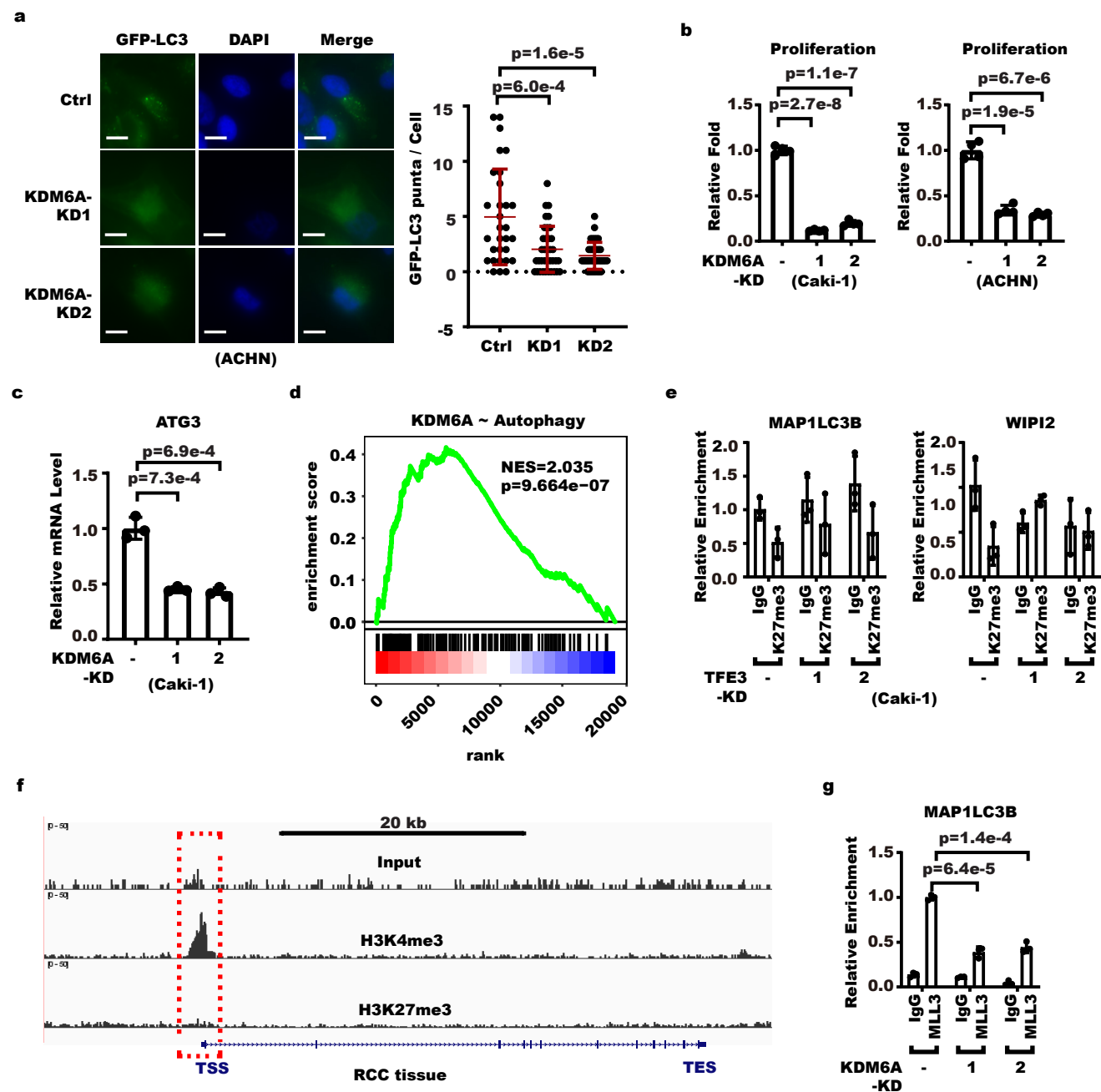

Supplement: Supporting Figure S8 [file mmc9.pdf]
