# Supplementary material for: TRIM28 represses renal cell carcinoma cell proliferation by inhibiting TFE3/KDM6A-regulated autophagy
Source: J Biol Chem. 2023 Mar 18;299(5):104621. doi: 10.1016/j.jbc.2023.104621 (PMC10141522; doi:10.1016/j.jbc.2023.104621)
Supplement: Supporting Figure Legends S1–S8 [file mmc10.docx]

**Supplementary Figure S1. Related to Figure 1**

**a.** Kaplan-Meier plot for survival of renal cell carcinoma patients with high (red, n=129) versus low (blue, n=129) TRIM28 mRNA level (data source: TCGA)

**b.** Kaplan-Meier plot for survival of renal cell carcinoma patients with high (red, n=58) versus low (blue, n=52) TRIM28 protein expression. (data source: CPTAC)

**c.** WB results for WCE of indicated cell lines of human kidney origin.

**Supplementary Figure S2. Related to Figure 2**

**a.** WCE of control and TRIM28-OE ACHN cells were analyzed with WB.

**b.** Myc-TRIM28 was overexpressed in ACHN cells stably expressing GFP-LC3. WCE were analyzed with WB.

**c.** Control and TRIM28-OE Caki-1 cells were analyzed with transmission electron microscopy (scale bar=200nm).

**d.** Control and TRIM28-OE Caki-1 cells were immunostained with LAMP1 antibody. Nuclei were counter-stained with DAPI. Shown on the left are photos from fluorescent microscopy (scale bar=10μm). Inserts are magnified as indicated (scale bar=1μm). Shown on the right is statistical analysis for LAMP1 puncta size with mean and standard deviation shown in red horizontal bars (684 puncta in control, 843 puncta in TRIM28-OE). P value was calculated from Student's t-test.

**e.** Myc-TRIM28 was overexpressed in ACHN cells. WCE were analyzed with WB. The left panel shows WB images. The right panel shows densitometry analysis of relative p-S6K level from three biological replicates. p-S6K level is presented as relative ratio to ACTIN. Error bars denote standard deviation and P value was calculated from Student's t-test.

**Supplementary Figure S3. Related to Figure 3**

**a.** WCE of control and TRIM28-KD Caki-1 cells were analyzed by WB.

**b.** WCE of control and TRIM28-OE Caki-1 cells were analyzed by WB.

**c.** Control or TRIM28-KD Caki-1 cells were analyzed by realtime RT-PCR. Shown are the relative mRNA levels of indicated genes. Error bars denote standard deviation of three technical replicates. P values were calculated from Student's t-test.

**d.** FLAG-TFE3 and Myc-TRIM28 were cotransfected into 293T cells. Cells were collected 48 hours later for coIP-WB analysis as indicated. "Input" denotes 1% input.

**e.** Interaction between endogenous TRIM28 and TFE3 in Caki-1 was analyzed with CoIP-WB assay. "Input" denotes 1% input.

**f.** WCE of control and TRIM28-KD ACHN cells were analyzed by WB.

**g.** Equal amounts of WCE from four cell lines were analyzed with WB.

**h.** TRIM28 was overexpressed in ACHN cells. TFE3 enrichment on WIPI2 gene promoter was then analyzed with ChIP followed with realtime-PCR. Error bars denote standard deviation of three technical replicates. P values were calculated from Student's t-test.

**i.** TFE3 was knocked-down in control or TRIM28-OE Caki-1 cells. Shown are real-time RT-PCR results for relative mRNA level of MAP1LC3B. Error bars denote standard deviation of three technical replicates. P values were calculated from one-way ANOVA and corrected for multiple comparison.

**j.** GSEA analysis for correlation between TRIM28 expression and autophagic gene signature in TCGA RCC patient samples in which TFE3 mRNA level is relatively higher than TFEB and MITF (64 patients for 1.5 fold higher, 19 patients for 2 fold higher).

**Supplementary Figure S4. Related to Figure 4**

**a.** TRIM28 was knocked-down in Caki-1 cells. Cells were then analyzed with realtime RT-PCR. Shown are the relative mRNA level of TFE3. Error bars denote standard deviation of three technical replicates.

**b.** Control and TRIM28-KD cells were treated with 25μg/ml Chx for indicated time. WCE were analyzed with WB. On the left shows blot images. On the right shows densitometry analysis of TFE3 level presented as relative ratio to ACTIN. Error bars denote standard deviation of three biological replicates. P values were calculated from two-way ANOVA.

**c.** Caki-1 and ACHN cells were treated with 25μM MG132 or 20μM HCQ for 8 hours. WCE were analyzed with WB.

**d.** Control or TRIM28-KD ACHN cells were treated with 25μM MG132 for 8 hours. WCE were analyzed with WB. On the left shows blot images. On the right shows densitometry analysis of TFE3 level presented as relative ratio to ACTIN. Error bars denote standard deviation of three biological replicates. P values were calculated from one-way ANOVA.

**e.** FLAG-TFE3, HA-SUMO1 and Myc-TRIM28 (WT for wildtype, C/A for C65A/C68A) were co-transfected into 293T cells as indicated. 48 hours later, cells were collected for IP-WB analysis as indicated.

**f**. TRIM28 Wild-type (WT) or inactive C65A/C68A mutant (C/A) was overexpressed in ACHN cells. WCE were analyzed with Western Blot.

**g.** Proliferation of the same cells as in (f) was analyzed with cell counting. Shown are relative proliferation fold over 7 days. Error bars denote standard deviation of four biological replicates. P values were calculated from one-way ANOVA.

**h.** 1000 TRIM28 Wild-type (WT) or inactive C65A/C68A mutant (C/A) cells were seeded into 3.5cm dishes. 14 days later, cell colonies were fixed and stained with crystal violet.

**Supplementary Figure S5. Related to Figure 5**

**a.** Myc-TRIM28 was expressed in Caki-1 cells. Cells were then subject to immunostaining with Myc-Tag antibody and nuclei were counter-stained with DAPI. On the left are photos from fluorescent microscopy (scale bar=10μm). On the right shows summary of cytoplasm/nucleus ratio of Myc-tag signal with mean and standard deviation shown in red horizontal bars. (n=31 cells for Caki-1, 22 cells for ACHN)

**b.** Summary of TRIM28 immunohistochemistry data in RCC patient tissues (Ab1 denotes antibody 1, Ab2 denotes Antibody 2, data source: The Human Protein Atlas).

**c.** TFE3 was knocked-down in 786-O RCC cells. Cells were then immunostained with TFE3 antibody. Nuclei were counter-stained with DAPI (scale bar=20μm). On the right shows summary of cytoplasm/nucleus signal ratio of TFE3 with mean and standard deviation shown in red horizontal bars (n=26 cells in ctrl).

**d.** Myc-TFE3 was overexpressed in Caki-1 or ACHN cells. Cells were then treated with EBSS (Earle’s Balanced Salt Solution) or 1μM TORIN1 for 4 hours. Afterwards, cells were immunostained with Myc-tag antibody with nuclei counter-stained with DAPI. On the left are photos from fluorescent microscopy (scale bar=10μm). On the right shows summary of cytoplasm/nucleus ratio of Myc-tag signal in each cell with mean and standard deviation shown in red horizontal bars (For Caki-1, n= 20 cells for "ctrl", 24 cells for "EBSS", 20 cells for "TORIN1"; For ACHN, n=10 in "ctrl", 16 in "EBSS", 18 in "TORIN1").

**e.** Normal kidney, liver and lung from BALB/c mouse were immunostained with TFE3 antibody with nuclei counter-stained with DAPI. Shown are photos from fluorescent microscopy (scale bar=20μm). For clarity, inserts framed in white were further magnified and shown on the right (scale bar=4μm).

**f.** Summary of TFE3 immunohistochemistry data in RCC, pancreatic cancer and lung cancer patient tissues. "None" means no signal was detected, "C" means mainly cytoplasmic signal, "N+C" means signal was in both cytoplasm and nuclei, "N" means mainly nuclear signal. P values were calculated from fisher exact test (n=12 in kidney cancer, n=12 in lung cancer, n=11 in pancreas cancer. Data source: The Human Protein Atlas).

**Supplementary Figure S6. Related to Figure 6**

**a.** Proliferation of control and TFE3-KD ACHN cells was analyzed with cell counting. Shown is relative proliferation fold over 7 days. Error bars denote standard deviation of four biological replicates. P values were calculated from Student's t-test.

**b.** 1000 control and TFE3-KD ACHN cells were seeded into 3.5cm dishes. 14 days later, cell colonies were stained with crystal violet.

**c.** Kaplan-Meiser plot for survival of RCC patients with low (blue) versus high (red) TFE3 protein level (58 patients in 'low', 52 patients in 'high', data source: CPTAC).

**d.** TFE3 was knocked-down in ACHN cells stably-expressing GPF-LC3. Nuclei were stained with DAPI. On the left shows photos from fluorescent microscopy (scale bar=10μm). Shown on the right is summary of GFP-LC3 puncta number per cell (n=26 cells for Ctrl, 28 cells for KD1, 24 cells for KD2). P values were calculated from Student's t-test.

**e.** Control and TFE3-KD Caki-1 cells were analyzed with transmission electron microscopy. Shown are photos of lysosomes (scale bar=200nm).

**f.** TFE3 was knocked-down in Caki-1 cells. Cells were then stained with LAMP1 antibody and nuclei were counter-stained with DAPI. On the left are photos from fluorescent microscopy (scale bar=10μm). Inserts framed in white were further magnified as shown (scale bar=1μm). On the right is summary of LAMP1 puncta size with mean and standard deviation shown in red horizontal bars (1488 puncta in ctrl, 3519 puncta in KD cells). P value was calculated from Student's t-test.

**g.** GSEA analysis for correlation between TFE3 mRNA level and autophagic gene signature in RCC patients from TCGA (532 patients in total, 64 patients for TFE3>(TFEB+MITF)>1.5).

**Supplementary Figure S7. Related to Figure 7**

**a.** Shown is profile plot for enrichment of KDM6A and HA-TFE3 at a metagene from ChIP-Seq data (all genes artificially resized to 10kB). TSS denotes transcription start site, TES denotes transcription termination site (data source: GEO GSE135490 and GSE172141).

**b.** Snapshot of UCSC Genome Browser shows enrichment of KDM6A, H3K4me3 and HA-TFE3 at WIPI2 gene locus (data source: ChIP from GEO GSE135490 and GSE172141).

**Supplementary Figure S8. Related to Figure 8**

**a.** KDM6A was knocked-down in ACHN cells stably-expressing GFP-LC3. Nuclei were counter-stained with DAPI. Shown on the left are photos taken from fluorescent microscopy (scale bar=10μm). Shown on the right is statistical analysis for GFP-LC3 puncta number in each cell (n=30 cells for Ctrl, 36 cells for KD1 and KD2) with mean and standard deviation shown as red horizontal bars. P values were calculated from Student's t-test.

**b.** Proliferation of control or KDM6A-KD Caki-1 and ACHN cells was measured by cell counting. Shown are relative proliferation fold over 9 days. Error bars denote standard deviation of four biological replicates. P values were calculated from Student's t-test.

**c.** Control or KDM6A-KD Caki-1 cells were analyzed with realtime RT-PCR. Shown are relative mRNA level of ATG3. Error bars denote standard deviation of three technical replicates. P values were calculated from Student's t-test.

**d.** GSEA for correlation between KDM6A expression and autophagic gene signature in 19 RCC patient samples where TFE3 mRNA level is higher than (TFEB+MITF) *2. (data source: TCGA).

**e.** H3K27me3 enrichment on WIPI2 promoter was analyzed with ChIP followed by realtime PCR in control and TFE3-KD Caki-1 cells. Error bars denote standard deviation of three technical replicates.

**f.** H3K4me3 and H3K27me3 at WIPI2 gene locus in human RCC tissue samples were examined by ChIP-Seq. Representative result from one patient is shown as snapshot of Integrative Genomic Viewer (data source: ChIP-Seq from GEO GSE75597).

**g.** MLL3 enrichment on MAP1LC3B promoter was analyzed with ChIP followed by realtime PCR in control and KDM6A-KD Caki-1 cells. Error bars denote standard deviation of three technical replicates. P values were calculated from Student's t-test.
